# Near-infrared co-illumination of fluorescent proteins reduces photobleaching and phototoxicity

**DOI:** 10.1038/s41587-023-01893-7

**Published:** 2023-08-03

**Authors:** Lucie Ludvikova, Emma Simon, Mathieu Deygas, Thomas Panier, Marie-Aude Plamont, Jean Ollion, Alison Tebo, Matthieu Piel, Ludovic Jullien, Lydia Robert, Thomas Le Saux, Agathe Espagne

**Affiliations:** 1 PASTEUR, Département de chimie, École normale supérieure, PSL University, Sorbonne Université, CNRS, Paris, France; 2 Institut Curie, Paris Sciences et Lettres (PSL) Research University, Centre National de la Recherche Scientifique (CNRS), Paris, France; 3 Institut Pierre-Gilles de Gennes, PSL Research University, Paris, France; 4 Sorbonne Université, CNRS, Institut de Biologie Paris-Seine (IBPS), Laboratoire Jean Perrin (LJP), Paris, France; 5 SABILab, Die, France; 6 Université Paris-Saclay, INRAE, AgroParisTech, Micalis Institute, Jouy-en-Josas, France

**Keywords:** Fluorescent proteins, Fluorescence imaging, Wide-field fluorescence microscopy, Photochemistry

## Abstract

Here we present a method to reduce the photobleaching of fluorescent proteins and the associated phototoxicity. It exploits a photophysical process known as reverse intersystem crossing, which we induce by near-infrared co-illumination during fluorophore excitation. This dual illumination method reduces photobleaching effects 1.5–9.2-fold, can be easily implemented on commercial microscopes and is effective in eukaryotic and prokaryotic cells with a wide range of fluorescent proteins.

## Abstract (web summary)

A dual illumination method reduces photobleaching for green and yellow fluorescent proteins.

## Main

The illumination required to excite fluorophores leads to their gradual photodegradation, a phenomenon known as photobleaching. Photobleaching limits the duration and time resolution of experiments and degrades image quality. Although its mechanisms are not fully understood, photobleaching is often assumed to involve the triplet excited state of the fluorophore and its interaction with molecular oxygen, leading to the formation of reactive oxygen species (ROS), which further react with the fluorophore^1–3^. The ROS resulting from fluorophore excitation are, in addition, harmful to cells and contribute to phototoxicity^4^.

This picture has inspired several strategies to reduce photobleaching and phototoxicity. Changes in the fluorophore environment, such as oxygen removal, the addition of antioxidants or the use of mixtures of reductants and oxidants to quench the triplet state, have been reported to be helpful^5–9^. These methods are easy to implement and compatible with any imaging technique, but they can have a strong impact on cellular physiology. Other approaches rely on spatial or temporal modulation of the intensity of the (one-color) excitation^10,11^, but they are specific to laser scanning microscopies, and their implementation is complex and expensive.

**Fig. 1:**
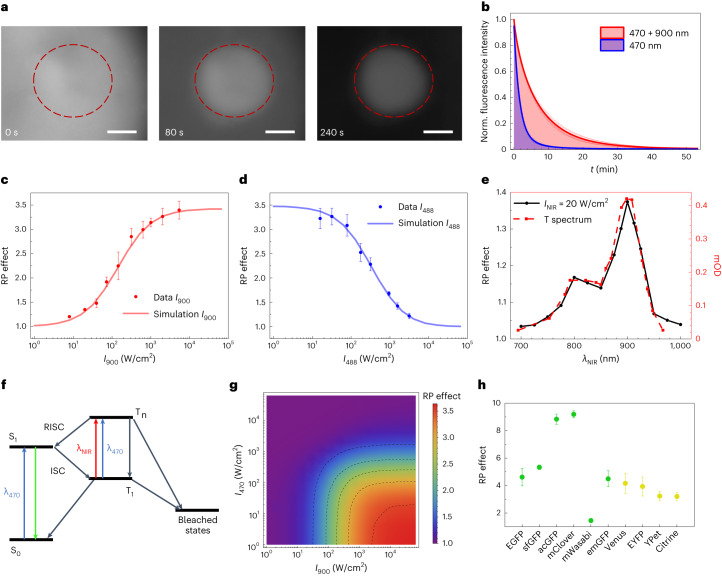
Mechanistic investigation of reduced photobleaching. **a**, Fluorescence images of EGFP immobilized in a PAA gel and illuminated at 470 nm (32 W/cm^2^, whole field) and at 900 nm (2 kW/cm^2^, red dashed circle). Scale bars, 20 µm. Representative of *n* = 4 replicate experiments. **b**, Photobleaching kinetics of EGFP illuminated solely at 470 nm (blue) or at both 470 nm and 900 nm (red). Values are mean ± s.d. (*n* = 4 samples). **c**,**d**, Dependence of the reduced photobleaching (RP) effect on NIR light intensity *I*_*900*_ (**c**; *I*_*470*_ = 32 W/cm^2^) and on visible light intensity *I*_*488*_ (**d**; *I*_*900*_ = 2 kW/cm^2^). Values are mean ± s.d. (*n* = 3 samples). Lines are simulations based on the model shown in **f**. **e**, Dependence of the RP effect on the NIR wavelength *λ*_*NIR*_. Black line: measurement performed with *I*_*470*_ = 32 W/cm^2^ and *I*_*NIR*_ = 20 W/cm^2^. Red dashed line: EGFP triplet absorption spectrum from ref. ^15^. mOD stands for 10^−3^ OD (optical density). **f**, Photophysical model. Triplet T_1_, a precursor of bleached states, forms from the bright S_1_ state under 470-nm illumination. NIR co-illumination promotes T_1_ into the higher triplet T_n_ from which the fluorophore returns to S_1_ by RISC. **g**, RP effect predicted by the model as a function of *I*_*470*_ (left axis) and *I*_*900*_ (bottom axis). **h**, RP effect for different green and yellow FPs expressed in *E. coli*. *I*_*470*_ = 32 W/cm^2^; *I*_*900*_ = 2 kW/cm^2^. Values are mean ± s.d. (*n* = 3 samples).

In this study, we explored a different strategy that is easy to implement and causes minimal perturbations to biological samples, namely the use of two-color illumination. It is known that the relaxation of the triplet states of some dyes can be accelerated by light through a photophysical process called reverse intersystem crossing (RISC)^12–14^. RISC is achieved by exciting the dye from its lowest triplet state to a higher triplet state from which it transitions back to singlet excited states before relaxing to the ground state. After the publication of the enhanced green fluorescent protein (EGFP) triplet absorption spectrum, which shows a maximum at 900 nm^15^, and the report of RISC in some yellow variants^14,16^, we hypothesized that the photobleaching of fluorescent proteins (FPs) under visible light excitation would be reduced by co-illumination with near-infrared (NIR) light. To assess this hypothesis, a sample of purified EGFP immobilized in a polyacrylamide (PAA) gel and continuously illuminated at 470 nm (32 W/cm^2^) was co-illuminated at 900 nm (2 kW/cm^2^) within a subregion of the field of view. After a few minutes, only the co-illuminated region was still visible (Fig. 1a). We measured a reduced photobleaching (RP) effect of 3.5, defined as the ratio of time-integrated emission in the presence of NIR light to the emission in absence of NIR light (Fig. 1b).

To specify the illumination conditions eliciting the RP effect, we next carried out a series of experiments on EGFP-PAA gels by varying the wavelength of the NIR laser and the intensities of the visible and NIR illuminations. The RP effect increased with NIR light intensity up to ~2 kW/cm^2^ where it reached a plateau (Fig. 1c) and decreased for visible light intensities higher than ~100 W/cm^2^ (Fig. 1d). The action spectrum at low NIR light intensity was found identical to the published triplet absorption spectrum (Fig. 1e), which proves both the involvement of the triplet in a photobleaching pathway of EGFP and the existence of RISC in this FP.

To understand further the mechanisms underlying the RP effect, we constructed a photophysical model describing the transitions between the relevant singlet and triplet states of EGFP (Supplementary Discussion). To account for the decrease of the RP effect at high visible light intensity and its saturation at high NIR light intensity, we had to extend the classical RISC model and consider the non-zero absorption of the lowest triplet state (T_1_) at 470 nm and a second photobleaching pathway from the upper triplet state (T_n_; Fig. 1f). This model enabled us to satisfactorily simulate the experimental intensity dependencies (Fig. 1c,d). It predicts a maximal RP effect (≥3) for EGFP when visible intensity is lower than 100 W/cm^2^ and NIR intensity is higher than 500 W/cm^2^ (Fig. 1g).

Then, we assessed the generality of the RP effect among FPs. NIR co-illumination had a beneficial effect for all green and yellow FPs tested (Fig. 1h and Supplementary Fig. 1), with an RP effect ranging from 1.5 to 9.2, likely reflecting slight variations in the photophysical parameters (Supplementary Discussion). The cyan variant ECFP showed, in contrast, no effect in the 700–1,000-nm range (Supplementary Fig. 2), whereas red FPs exhibited a more complex behavior (Supplementary Fig. 3).

**Fig. 2:**
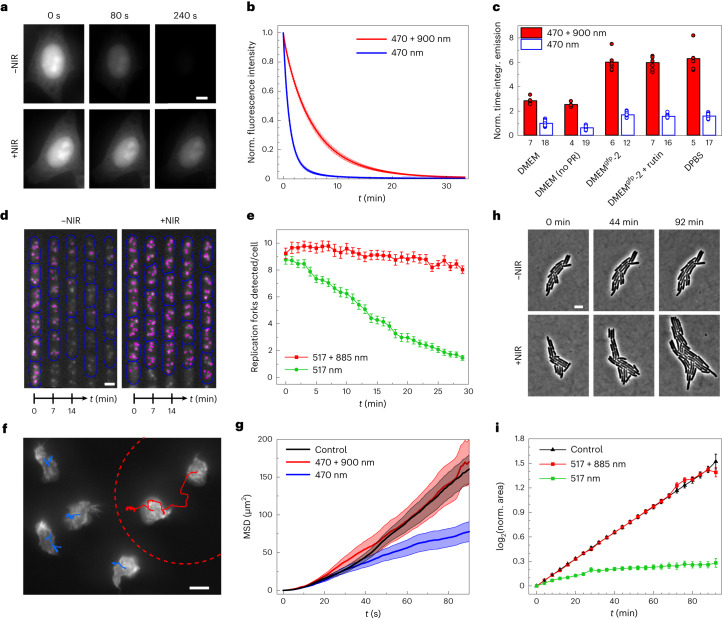
NIR co-illumination reduces photobleaching and phototoxicity in eukaryotic and prokaryotic cells. **a**, Fluorescence images of fixed HeLa cells labeled with EGFP and illuminated at 470 nm alone (top) or combined with 900 nm (bottom). Scale bar, 10 µm. Representative of *n* = 3 replicate experiments. **b**, Photobleaching kinetics of fixed HeLa cells. Values are mean ± s.d. (*n* = 12 cells examined over three samples). **c**, Time-integrated emission of live HeLa cells labeled with EGFP and illuminated at 470 nm alone (white bars) or combined with 900 nm (red bars), in different media. PR, phenol red. All integrals were normalized to that measured in standard DMEM in the absence of NIR light. Dots correspond to individual cells and bar heights to mean. The number of cells is indicated below the bars. **d**, Kymographs of *E. coli* bacteria (blue contours) expressing the DnaN-YPet fusion to mark replisomes (magenta spots). Bacteria growing in the ‘mother machine’ microfluidic chip at 37 °C were imaged in fluorescence in three *z* planes distant from 350 nm by illumination at 517 nm alone (left) or combined with 885 nm (right). We show the middle plane with replisomes detected by exploiting the three planes. Scale bar, 2 μm. Representative of *n* = 2 replicate experiments. **e**, Number of replisomes detected per cell as a function of time. Values are mean ± s.e.m. (*n* = 60 cells). **f**, Fluorescence image with 5-min cell tracks of primary mouse neutrophils expressing LifeAct-GFP and migrating in a PDMS microchamber at 37 °C under illumination at 470 nm (whole field) and 900 nm (red dashed circle). Scale bar, 10 μm. Representative of *n* = 2 replicate experiments. **g**, Mean squared displacement (MSD) of neutrophils. The control was obtained using minimal 470-nm illumination. Values are mean ± s.e.m. (*n* = 32 cells for 470-nm illumination, *n* = 14 cells for dual illumination and *n* = 46 cells for control). **h**, Phase contrast images showing the growth of YPet-labeled *E. coli* microcolonies on an agarose pad at 37 °C under illumination at 517 nm alone (top) or combined with 885 nm (bottom). Scale bar, 5 µm. Representative of *n* = 2 replicate experiments. **i**, Time evolution of the binary logarithm of the area of the bacterial colonies normalized to their initial area. Values are mean ± s.e.m. (*n* = 14 colonies for 517-nm and dual illumination and *n* = 4 colonies for control, examined over two samples). All illumination conditions are specified in Supplementary Table 1.

Strong RP effects obtained for moderate intensities of visible light suggested that NIR co-illumination could be used to reduce FP photobleaching in live cell imaging using wide-field microscopy (typical intensities ≤1 W/cm^2^). As a control, we first performed in situ temperature measurements with a molecular probe^17^ and found no significant temperature increase due to NIR light (Supplementary Fig. 4). Next, we implemented NIR co-illumination on a Nikon Eclipse Ti wide-field fluorescence microscope. A straightforward modification of the epifluorescence illuminator to integrate an 885-nm laser diode enabled us to achieve an NIR co-illumination of 0.8 kW/cm^2^ over the same field as the visible excitation (Supplementary Fig. 5). As expected, using this setup, we observed a strong RP effect for EGFP and the yellow FP YPet (Supplementary Fig. 6). We then evaluated the effect of NIR light on FP-labeled biological samples. NIR co-illumination of EGFP expressed in live or fixed HeLa cells or in live bacteria induced a reduction in photobleaching similar to that obtained in vitro (Fig. 2a,b, Supplementary Fig. 7 and Supplementary Video 1). By comparing the photobleaching kinetics of live HeLa cells labeled with EGFP in different media, we found, in addition, that NIR co-illumination in standard DMEM increases the time-integrated emission more than exchanging the medium against vitamin-depleted DMEM^gfp^-2 (refs. ^8,9^), which is currently the main available solution to reduce FP photobleaching in live mammalian cells. Both strategies can be combined to obtain an even higher six-fold increase of emission (Fig. 2c and Supplementary Fig. 8).

To further demonstrate that our method can substantially improve in vivo wide-field imaging, we followed fluorescently tagged replisomes (DnaN-YPet) in *Escherichia coli* cells in fast growth conditions (see Methods for details). Many recent studies on the bacterial cell cycle involving replisome visualization have been hampered by the difficulty to detect and track the numerous replisomes present in fast-growing cells^18,19^. Accurate detection and tracking requires imaging several *z* planes at high temporal resolution, which leads to substantial photobleaching of the FP (Supplementary Fig. 9). We found that NIR co-illumination greatly prolongs the visualization of replisomes. As expected, YPet photobleaching under classical one-color excitation led to a rapid decrease in the number of replisomes detected by our analysis software. In contrast, NIR co-illumination enabled us to detect a constant number of ~9 replisomes per cell throughout the cycle (Fig. 2d,e and Supplementary Video 2).

We next reasoned that, by quenching the fluorophore triplet state, NIR co-illumination should also decrease ROS production and, hence, phototoxicity, and we tested this hypothesis in mammalian cells and bacteria. Mammalian cell migration has been shown to be a sensitive readout of phototoxicity^20^. We examined the motility of primary mouse neutrophils expressing LifeAct-GFP under different illumination conditions. Cells illuminated with 20 W/cm^2^ of 470-nm light traveled shorter trajectories than minimally illuminated control cells, showing the phototoxic effect of such illumination. As expected, this phototoxicity was substantially reduced by NIR co-illumination (Fig. 2f,g, Supplementary Fig. 10 and Supplementary Video 3). In addition, NIR light led to a substantial reduction of GFP photobleaching (Supplementary Fig. 10). As a second assay, we monitored the growth of FP-labeled *E. coli* microcolonies in the presence and absence of NIR light. Cell proliferation is the standard measure of fitness in bacteria and a well-established criterion for assessing phototoxicity^4^. NIR co-illumination had no effect on phototoxicity for EGFP-labeled bacteria excited at 490 nm, likely because toxicity at this wavelength is mainly caused by endogenous photosensitizers (Supplementary Fig. 11 and Supplementary Note 1). In contrast, we found that NIR co-illumination substantially reduces phototoxicity for YPet-labeled bacteria illuminated at 517 nm. Depending on the exposure time, 517-nm illuminated bacteria hardly grew (Fig. 2h,i and Supplementary Video 4) or grew slower than the non-illuminated control (Supplementary Fig. 12), whereas dual illumination at 517 nm and 885 nm restored a normal growth rate. As expected, FP photobleaching was also substantially reduced by NIR co-illumination in these experiments (Supplementary Fig. 13).

In conclusion, this work shows that the photophysical process of RISC can be exploited to reduce the photobleaching and phototoxicity of FPs in vivo in wide-field imaging, through NIR co-illumination at ~900 nm. This approach can be easily implemented on commercial microscopes, by adding a light source at ~900 nm. Unlike anti-fading reagents and media, it does not require any special preparation of samples before imaging nor the use of a particular culture medium, which makes it compatible with a wide range of biological samples. Beyond wide-field imaging, such NIR co-illumination of FPs could be beneficial for all imaging techniques using visible excitation intensities lower than ~100 W/cm^2^.

## Methods

### Photophysical characterization of the RP effect

#### Plasmids for bacterial expression of FPs

The plasmid for bacterial expression of emGFP was purchased from Invitrogen (pRSET-EmGFP, ref. V35320). The genes encoding sfGFP, acGFP, mWasabi and mClover were ordered from Integrated DNA Technologies and inserted into the pET28a bacterial expression vector (Novagen) by Gibson assembly using appropriate primers. See Supplementary Table 2 for the sequences of the g-blocks and primers used. The constructs for bacterial expression of EGFP, Venus and mCherry, based on the same pET28a vector, are gifts from Arnaud Gautier (Sorbonne Université) and are described in ref. ^21^. The constructs for bacterial expression of ECFP and EYFP are based on the pPROEX HTa vector (Invitrogen) and are gifts from Regis Grailhe (Institut Pasteur Korea). The constructs for bacterial expression of Citrine, YPet and mRFP1 are gifts from Robert Campbell, Michael Davidson, Oliver Griesbeck and Roger Tsien^22^, Patrick Daugherty and Michael Davidson^23^ and Philippe Cluzel^24^, respectively (Addgene plasmids 54772, 54860 and 104001).

#### EGFP expression and purification

His-tagged EGFP was expressed in *E. coli* BL21(DE3) (New England Biolabs) using the pET28a expression vector (Novagen). The bacteria were transformed by electroporation following a standard protocol. Then, 250 ml of TB-kanamycin (50 μg ml^−1^) was inoculated with an overnight preculture and grown at 37 °C, 220 r.p.m., until the optical density at 600 nm (OD_600_) reached 0.6 (2–4 h), and then IPTG (1 mM) was added, and the temperature was lowered to 16 °C. After overnight expression, the cells were collected by centrifugation (15 min, 4,700*g*), and then the pellet was washed with 6 ml of PBS and centrifuged again (10 min, 11,000*g*) and then frozen. The pellets were resuspended in lysis buffer (5 ml of PBS, 25 μl of 5 mg ml^−1^ DNase, 50 μl of 100 mM PMSF), sonicated for 5 min and incubated on ice for 2 h. The mixture was centrifuged (11,000*g*, 1 h, 4 °C) to remove cell debris, and the supernatant was incubated overnight at 4 °C with Ni-NTA agarose beads (Sigma-Aldrich) on a rotator mixer. Ni-NTA affinity chromatography was carried out using a Glass Econo-Column (Bio-Rad, 0.7 cm × 20 cm). The beads were washed with PBS buffers containing 30 mM, and then 10 mM imidazole and EGFP was finally eluted with PBS buffer containing 150 mM imidazole. Imidazole was removed by overnight dialysis against PBS buffer using Slide-A-Lyzer Dialysis Cassettes (Thermo Fisher Scientific). The concentration of EGFP solutions was calculated from their absorption spectrum measured on a Cary 300 Scan spectrophotometer (Varian) using a molar absorption coefficient of 55,900 mol^−1^ L cm^−1^ at 488 nm^25,26^. They were aliquoted and stored at −20 °C until use.

#### EGFP-PAA gels

Next, 21 μl of purified EGFP (40 μM) was mixed with 15 μl of 40% acrylamide/bis-acrylamide (37.5:1 ratio, Sigma-Aldrich) and 0.5 μl of 100× diluted fluorescent beads (FluoSpheres, 0.04 μm, yellow-green, Invitrogen) to facilitate focusing. Polymerization was initiated by 0.1% ammonium persulfate and 0.1% TEMED (*N*,*N*,*N*′,*N*′-tetramethylene-ethylenediamine). The mixture was placed on a coverslip, covered with another coverslip to form a uniform thin layer (∼30 µm) and left to cross-link into gel overnight at room temperature.

#### Bacteria samples for RP effect measurement in different FPs

One colony of *E. coli* BL21 cells expressing the FP of interest was picked from the Petri dish, added to 2 ml of LB-kanamycin (50 μg ml^−1^) and grown overnight at 37 °C and 220 r.p.m. Then, 3–10 μl of this preculture was spread on a PBS-agarose pad (1% agarose, thickness 0.25 mm) on a coverslip and covered with another coverslip. The samples were left to settle down overnight at room temperature before use.

#### Optical setup and data acquisition

Photophysical characterizations of the RP effect were performed on an in-house-built wide-field fluorescence microscope equipped with a ×40/0.75 numerical aperture (NA) air objective (Olympus). The 470-nm excitation was delivered over a 200-µm-diameter round spot by an LED at 470 ± 20 nm (Thorlabs, M470L4) filtered through a 479 ± 40-nm interference filter (BrightLine, Semrock) and the NIR co-illumination over a smaller 45-µm-diameter round spot by a laser tunable in the 700–1,000-nm range (Coherent, Mira, operated in continuous-wave mode) (Supplementary Fig. 14a). To measure the dependence of the RP effect on visible intensity, the LED was replaced by a 488-nm laser diode (Omicron, LuxX 488-100) that was overlapped with the NIR laser (45-µm-diameter round spot) to achieve intensities in the 31–5,388-W/cm^2^ range (Supplementary Fig. 14b). The emission light was spectrally filtered with an interference filter at 525 ± 30 nm (BrightLine, Semrock). Two dichroic mirrors, FF506-Di03 (BrightLine, Semrock) and 680dcspxr (Chroma, short-pass), were used to separate the emission from the visible and NIR illuminations. An additional short-pass filter (FF02-694/SP-25, Semrock) was placed in front of the camera to eliminate NIR light. The intensities of both illuminations were measured before and after each measurement using a power meter (Thorlabs). Fluorescence images were recorded with an IMX250 CMOS camera (iDS, UI-3080CP-M-GL Rev.2) controlled by µManager 2.0 software. Although both illuminations were continuous, images were taken every 8–60 s (exposure times, 15–50 ms) with the 470-nm LED as excitation source and every 10 ms to 12 s (exposure times, 0.5–15 ms) with the 488-nm laser diode.

#### Data analysis

The images were first analyzed with Fiji to extract the photobleaching kinetics. The instant fluorescence was taken as the average over selected regions of interest (ROIs). The data were further analyzed in OriginPro 2015. Data measured on FP-labeled cells were background subtracted to remove the contribution from medium autofluorescence, and the fluorescence intensities of all cells were normalized to 1 at *t* = 0. ROIs in EGFP-PAA gel samples were chosen with same initial intensity values. Unless otherwise mentioned, the magnitude of the RP effect was quantified as the ratio of the areas under the photobleaching curves measured with and without NIR co-illumination.

#### Simulations

Numerical simulations of the photobleaching kinetics of EGFP and the dependence of the RP effect on the intensity of the 470-nm and NIR illuminations, the wavelength of the NIR illumination and the values of different photophysical parameters were performed based on the model of Fig. 1f, using Wolfram Mathematica 12. A complete description of these simulations can be found in the Supplementary Discussion.

### Measurement of sample temperature under NIR illumination

The temperature in the presence of the NIR beam was measured using a DNA-based ratiometric fluorescent temperature probe^17^. A 2-mm-thick PDMS spacer (typical medium height in mammalian cell samples) was placed on a coverslip, filled with the probe solution (20 µM in 10 mM HEPES buffer pH 7.5 with 0.1 M NaCl and 2 mM MgCl_2_) and covered with another coverslip. A calibration curve was first measured in the absence of the NIR beam. The sample was placed on an aluminum block whose temperature was varied between 30 °C and 45 °C using thermoelectric Peltier devices (CP 1.0-63-05L-RTV, Melcor) driven by a MPT1000 temperature controller (Wavelength Electronics) equipped with a TCS610 thermistor (Wavelength Electronics). A 300-µm-thick copper slide with a 2-mm-wide and 3-cm-long slit for observation was coated with vacuum grease and slid between the aluminum block and the sample to improve thermal contact. The sample was excited at 470 nm and imaged in the green and red channels (ET525/36m (Chroma), resp. 628/32 BrightLine (Semrock)) so as to obtain the ratio of green intensity to red intensity as a function of temperature. The temperature of the heating stage was then set at 37 ± 0.2 °C, and new green and red images were measured in the presence of the NIR beam (80-μm diameter, 1.7 kW/cm^2^ at 900 nm) to obtain a temperature map. The data were analyzed using Fiji and MATLAB.

### HeLa cell experiments

The construct for mammalian expression of EGFP is described in ref. ^21^. The HeLa cells (from American Type Culture Collection cell line CCL-2) were cultured in DMEM supplemented with phenol red, GlutaMAX I and 10% (v/v) FCS. For microscopic imaging, cells were seeded in poly-lysine-coated 35-mm ibidi μ-dishes (5 × 10^5^ cells per milliliter) and transfected with 2 μg of plasmid DNA for EGFP expression using Lipofectamine 2000 according to the manufacturer’s instructions. After 24 h, the cells were washed twice with 1× DPBS, and the medium was exchanged to DMEM (without serum) for live cell imaging, or they were fixed for 20 min at room temperature with 2% paraformaldehyde. For the assessment of EGFP photostability in different media, the medium was exchanged 30 min before imaging, and the samples were left for that time in the incubator. The following media were compared: DMEM with phenol red (Gibco, Thermo Fisher Scientific, 11995065), DMEM without phenol red (Gibco, Thermo Fisher Scientific, 31053028), DPBS (10×, Gibco, Thermo Fisher Scientific, 14200075) and DMEM^gfp^-2 with and without rutin (Evrogen). Microscopy experiments on HeLa cells were performed with our in-house-built wide-field fluorescence microscope with dual illumination and analyzed with Fiji and OriginPro 2015 in the same way as in vitro experiments (see above).

### Neutrophil experiments

#### Neutrophil purification and culture

Mouse neutrophils were isolated from the bone marrow of healthy 12-week-old C57Bl6/J LifeAct-GFP mice using the MojoSort Mouse Neutrophil Isolation Kit (BioLegend) according to the manufacturer’s instructions. Freshly purified mouse neutrophils were cultured overnight in RPMI (Gibco) supplemented with 10% FBS (Euroclone), 50 ng ml^−1^ GM-CSF (Miltenyi Biotec) and 25 mM HEPES (Gibco) at 37 °C with a 5% CO_2_ atmosphere. For animal care, we strictly followed the European and French National Regulation for the Protection of Vertebrate Animals used for Experimental and other Scientific Purposes (Directive 2010/63; French Decree 2013-118). The present experiments, which used mouse strains exhibiting non-harmful phenotypes, did not require a project authorization and benefited from guidance of the Animal Welfare Body, Research Center, Institut Curie. Mice were housed under a 12:12 light/dark cycle in a pathogen-free facility with controlled temperature (22  ± 2 °C) and humidity (55% ± 10%). C57Bl6/J LifeAct-GFP mice were a kind gift from Ana Maria Lennon-Dùmenil (Institut Curie).

#### Preparation of microchambers

Neutrophil motility was assessed with the use of a specific microdevice consisting of a chamber of 4-µm height as previously described^27,28^. In brief, PDMS (RTV615, Neyco) was used to make microchambers from custom-made molds prepared using a standard photolithography technique. The PDMS chamber and a 35-mm glass-bottom dish (FD35-100, WPI) were plasma activated before being bound to each other. The microdevices were then plasma cleaned, coated with 10 μg ml^−1^ fibronectin (Sigma-Aldrich) for 30 min and then washed three times with PBS and incubated 2 h with complete media. Neutrophils were loaded (10^5^) in 3-mm holes punched before plasma treatment. The microdevice was gently flooded with complete media, and neutrophils spontaneously migrated in the 4-µm-height confined regions, where their motility was assessed 1 h later.

#### Data acquisition

Microscopy experiments on neutrophils were performed using our in-house-built fluorescence microscope with dual illumination (see above), equipped with a UI-3080CP-M-GL (IDS GmbH) or an Orca-Flash4.0 V3 (Hamamatsu Photonics KK) camera. The NIR beam, modulated with an SC10 mechanical shutter (Thorlabs), was expanded before being injected into the microscope objective to produce a homogeneous 80-µm-diameter spot at the sample that typically covered 2–3 neutrophils per field of view. The neutrophil dishes were thermostated at 37 °C ± 0.2 °C using the same heating stage as for temperature measurements. To avoid pH drift, cells observed for 30–60 min were returned to the CO_2_ incubator for 1–2 h before any re-observation. Cells were imaged for 5 min under weak (0.8 W/cm^2^, 200 ms of exposure every 1 s) or strong (20 W/cm^2^, 200–400 ms of exposure every 1 s) 470-nm excitation in the presence or absence of 900-nm light (2 kW/cm^2^, 200–400 ms of exposure every 1 s).

#### Neutrophils segmentation and tracking

Image analysis was performed using BACMMAN software^29^ (https://github.com/jeanollion/bacmman) that allows automatic cell segmentation and tracking as well as manual curation of both processes. We developed a method for segmentation using a deep neural network (DNN) trained on a very small training set of nine partially annotated images. Our method involves predicting three classes: background, contours and foreground. We found that predicting contours improved the precision of segmentation. See Supplementary Note 2 for details on DNN architecture, training parameters, training set and data augmentation. The cells were then segmented by applying a watershed transform to the predicted probability of the foreground class. Tracking was performed with BACMMAN by integrating the TrackMate^30^ implementation of the algorithm by Jaqaman et al.^31^, which employs the Hungarian algorithm to solve the linear assignment problem.

The tracking data were analyzed using Python. Cells that left the visible or NIR field during tracking were excluded from the analysis, as were overlapping cells.

### Bacterial growth experiments

#### Sample preparation

The strains used were *E. coli* BL21 expressing EGFP and *E. coli* MG1655 (CGSC 6300) either unlabeled or expressing YPet. All incubation steps were performed at 37 °C under agitation. Cultures were inoculated in LB broth, supplemented with ampicillin (100 μg ml^−1^) for MG1655-YPet or kanamycin (50 μg ml^−1^) for BL21-EGFP and incubated overnight. Before plating on a microscope slide, cultures were prepared as follows. Cultures of BL21-EGFP were diluted 200-fold in LB supplemented with kanamycin (50 μg ml^−1^) and IPTG (100 µM), incubated for 2 h and diluted again 200-fold. Cultures of MG1655 were diluted 200-fold in LB, incubated for 1.5 h and diluted again 200-fold. Cultures of MG1655-YPet were diluted 1,000-fold in M9 Minimal Medium (M9 salts 5× from Sigma-Aldrich diluted to a 1× working solution and supplemented with 2 mM MgSO_4_) containing casamino acids (0.2%), arabinose (0.2%) and ampicillin (100 μg ml^−1^) (MMcasa medium), incubated for 3 h and then diluted again four times. The slides were prepared using the following protocol. Low-melting-temperature agarose (20 g L^−1^) was dissolved into the medium (MMcasa for MG1655-YPet, LB for MG1655, LB supplemented with 50 μg ml^−1^ kanamycin and 100 µM IPTG for BL21-EGFP) by heating. A Gene Frame (Thermo Fisher Scientific) was stuck on a glass slide, and the resulting cavity was filled with the medium containing agarose, covered with a microscope slide and cooled for 2–3 h at room temperature. Then, the slide was removed, and stripes of agarose were removed using a surgical scalpel to leave one small stripe (~5 mm wide), surrounded by air cavities ensuring oxygenation. Then, 2 μl of the bacterial culture was deposited on the slide. Once the liquid was absorbed, the cavity was sealed with a coverslip, and the slide was incubated at 37 °C for several hours before imaging (~2 h for cells growing in LB and ~3 h for cells growing in MMcasa), allowing cells to grow into small microcolonies.

#### Optical setup, data acquisition and analysis

The bacterial growth experiments were performed with a Nikon Eclipse Ti microscope controlled by NIS software, whose epifluorescence illuminator was modified to deliver dual visible and NIR illumination (Supplementary Fig. 5). In brief, the NIR light source was a fibered 1-W, 885-nm diode laser (RLTMDL-885-1W, Roithner), and the visible light source was an LED (M490L4 or M530L4, Thorlabs). After collimation of the two beams and expansion of the NIR beam, they were combined using a short-pass dichroic mirror (DMSP805R, Thorlabs) inserted in a filter cube, which replaced the main body of the illuminator. To reflect both the NIR and visible beams to the sample and collect the fluorescence signal from EGFP or YPet, we equipped a filter cube of the microscope turret with appropriate combinations of a dichroic mirror and a band-pass emission filter (T495lpxr + ET525/50m for EGFP or ZT514rdc + ET545/40m for YPet, all from Chroma). We placed an additional short-pass filter (FESH0650, Thorlabs) below the filter turret to protect the camera from residual NIR light. The objective was a Plan APO ×100 immersion objective (NA 1.4). To assess phototoxicity and photobleaching, growing microcolonies either unlabeled or expressing EGFP or YPet were exposed to blue light (490 nm, 0.5 W/cm^2^) or green light (517 nm, 1.2 W/cm^2^), respectively, in the presence or absence of NIR co-illumination (885 nm, 0.8 kW/cm^2^). Their growth at 37 °C was followed by phase contrast imaging and fluorescence imaging (for FP-labeled bacteria). The data were analyzed with Fiji and OriginPro 2015 in the same way as in vitro data (see above). The areas of the microcolonies were extracted from phase contrast images using Fiji.

### Experiments on replisomes

The strain was obtained by transduction of the YPet-DnaN allele^32^ (fluorescent fusion of the β-subunit of the DNA polymerase, kind gift from Sven van Teeffelen) into *E. coli* MG1655. An overnight culture in LB medium was loaded into the ‘mother machine’ microfluidic chip^33^. Fabrication of the chip and cell loading are explained in detail in ref. ^34^. A flow of 2 ml h^−1^ of LB medium supplemented with 0.1 mg ml^−1^ BSA was delivered to the cells using a PHD ULTRA syringe pump (Harvard Apparatus), allowing fast growth of the cells (~22 min doubling time; an average number of ~10 replisomes per cell is expected in these conditions^35^). The chip was installed on our Nikon Eclipse Ti microscope, modified for dual illumination as explained above and thermostated at 37 °C. Image acquisition began after 4 h of incubation, when cells had reached balanced, exponential growth. Three images corresponding to three different focal planes, distant from 350 nm, were taken every minute, with 300-ms exposure time. Replisomes were segmented using BACMMAN software^29^ (https://github.com/jeanollion/bacmman) with the same method used to detect fluorescent foci as in ref. ^36^. This method detects spots on a criterion based on both the intensity value and the Laplacian transform value. We optimized thresholds so that spots are well detected at the first frame and used the same thresholds for the whole movie in both conditions with and without NIR.

### Reporting summary

Further information on research design is available in the Nature Portfolio Reporting Summary linked to this article.

## Online content

Any methods, additional references, Nature Portfolio reporting summaries, source data, extended data, supplementary information, acknowledgements, peer review information; details of author contributions and competing interests; and statements of data and code availability are available at 10.1038/s41587-023-01893-7.

### Supplementary information

Supplementary Information Supplementary Discussion, Supplementary Notes 1 and 2, Supplementary Figs. 1–14, Supplementary Tables 1 and 2 and captions of Supplementary Videos 1–4

Reporting Summary

Supplementary Video 1 NIR co-illumination reduces the photobleaching of live HeLa cells labeled with EGFP

Supplementary Video 2 NIR co-illumination extends the visualization of YPet-labeled replisomes in rapidly growing *E. coli* cells

Supplementary Video 3 NIR co-illumination reduces photobleaching and phototoxicity in primary mouse neutrophils expressing LifeAct-GFP

Supplementary Video 4 NIR co-illumination reduces phototoxicity in YPet-labeled *E. coli* cells

## Data Availability

The data that support the findings of this study are available at 10.5281/zenodo.8069922.

## Code availability

BACMMAN software used to analyze the replisome and neutrophil experiments is available at https://github.com/jeanollion/bacmman/. The code developed for neutrophil segmentation (deep learning model, training procedure and associated documentation) is available at https://github.com/jeanollion/pix_mclass.
