# Supplementary material for: Near-infrared co-illumination of fluorescent proteins reduces photobleaching and phototoxicity
Source: Nat Biotechnol. 2023 Aug 3;42(6):872–6. doi: 10.1038/s41587-023-01893-7 (PMC11180605; doi:10.1038/s41587-023-01893-7)
Supplement: Supplementary file 1 — Supplementary Discussion, Supplementary Notes 1 and 2, Supplementary Figs. 1–14, Supplementary Tables 1 and 2 and captions of Supplementary Videos 1–4 [file 41587_2023_1893_MOESM1_ESM.pdf]

nature biotechnology

Brief Communication

https://doi.org/10.1038/s41587-023-01893-7

Near-infrared co-illumination of fluorescent proteins reduces photobleaching and phototoxicity

In the format provided by the authors and unedited

Contents

1. Supplementary discussion 2
1.1. Photophysical model of the RP effect 2
1.2. Simulation of the RP effect in EGFP 4
1.3. Sensitivity of the RP effect to the fluorophore's photophysical parameters 9
2. Supplementary notes 13
3. Supplementary figures 14
4. Supplementary tables 30
5. Supplementary video captions 32

1. Supplementary discussion

1.1. Photophysical model of the RP effect

We constructed the simplest photophysical model accounting for the observed reduced photobleaching of EGFP under continuous dual visible and NIR illumination (Fig. 1a,b) and its dependence on the illumination intensity I_{NIR} in the NIR (Fig. 1c), the illumination intensity I_{470} at 470 nm (Fig. 1d) and the wavelength λ_{NIR} of the NIR illumination (Fig. 1e).

We first considered a basic RISC model (Supplementary Fig. SD1, black arrows). In this model, the EGFP chromophore is initially excited by absorption of a visible photon (wavelength 470 nm) from the ground-state S_0 to the highly fluorescent singlet excited state S_1 (**1**). The excited molecules which do not relax back to the ground state via fluorescence or internal conversion (**2**) evolve via intersystem crossing (ISC, **3**) to the longer-living triplet excited state T_1 which can undergo irreversible chemical changes to non-fluorescent (bleached) states D (**4**) or relax back to S_0 (**5**). Re-excitation of triplet T_1 by the NIR illumination (wavelength λ_{NIR}, **6**) to a higher-lying triplet state T_n decreases the photobleaching rate by depopulating T_1. Once formed, T_n can convert back to S_1 by reverse intersystem crossing (RISC, **7**) or relax to T_1 by internal conversion (**8**). The role of the triplet state T_1 as an intermediate in photobleaching and the existence of RISC in EGFP are both supported by the action spectrum of the RP effect (Fig. 1e) which is similar to the published absorption spectrum of the EGFP triplet.[1]

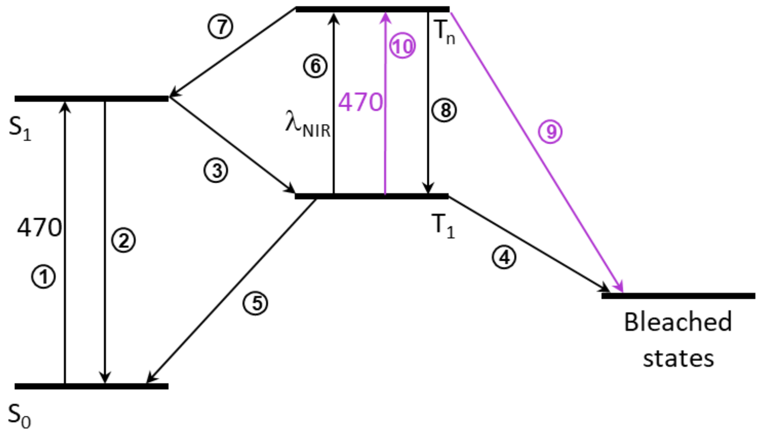

Supplementary Fig. SD1: Photophysical model of the RP effect (similar to Fig. 1f). Black arrows correspond to the basic RISC model while purple arrows show the additional processes we had to include to satisfactorily reproduce the experimental intensity-dependencies.

Simulations based on this basic model led however to a linear dependence of the RP effect on I_{900}, in contrast with experimental observations (Supplementary Fig. SD2, black line and red circles, resp.). To obtain a saturation of the RP effect at high I_{900} (Supplementary Fig. SD2, red line), we had to add a second photobleaching pathway starting from T_n (Supplementary Fig. SD1, **9**). Moreover, simulations based on a model including only processes **1** to **9** led to a RP effect independent of I_{470}, in disagreement with the data (Supplementary Fig. SD3, red line and blue circles, resp.). To reproduce the decrease of the RP effect at high I_{470} (Supplementary Fig. SD3, blue line), we had to take the non-zero absorption of T_1 at 470 nm into account (Supplementary Fig. SD1, **10**). The latter leads to saturation of the $T_1 \rightarrow T_n$ transition by 470-nm light at high I_{470}, so that the NIR co-illumination does not provide any additional benefit. Higher singlet S_p ($p > 1$) and triplet T_q ($q > n$) excited states were neglected in our model due to their fast depopulation (ps) to lower-lying states of the same spin multiplicity.

Our final model is close to that of Ringemann et al. who first came up with the idea of exploiting RISC in fluorophores, showing that the process can significantly increase the fluorescence signal of small organic fluorophores excited with very high intensities (0.1-10 MW/cm^2).[2] Due to our bottom-up approach of starting from a basic model and gradually adding photophysical processes until we account for the experimental intensity dependencies, and our much lower excitation intensities (1-10 W/cm^2 for visible excitation and up to a few kW/cm^2 for NIR co-illumination), our model is however simpler. In particular, it does not take singlet excited states above S_1 into account, nor photobleaching from S_1, nor stimulated emission, which could all be neglected in our experimental conditions.

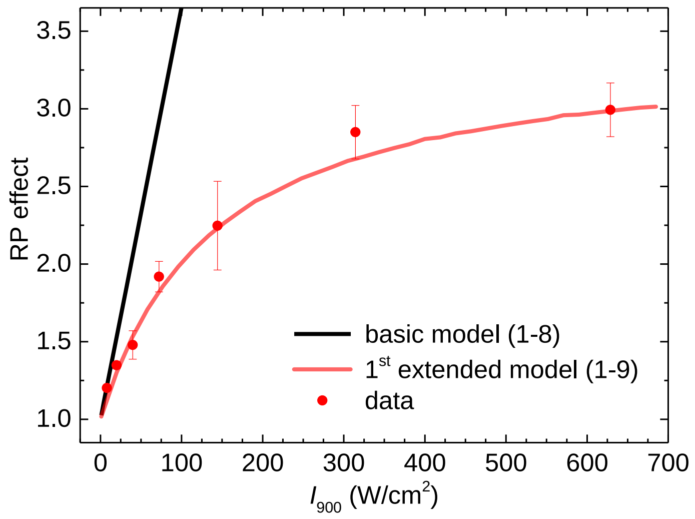

Supplementary Fig. SD2: Simulations of the expected RP effect dependence on 900-nm intensity for the basic RISC model including only processes **1-8** (black line) and for the first extended model including in addition photobleaching from T_n (process **9**; red line). Values are mean ± s.d. (n=3 samples).

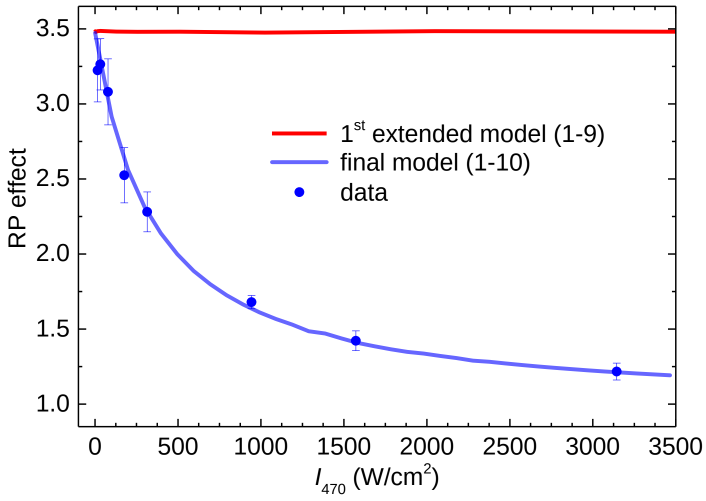

Supplementary Fig. SD3: Simulations of the expected RP effect dependence on 470-nm intensity for the first extended model including only processes **1-9** (red line) and for the final model including in addition T_1 absorption at 470 nm (process **10**; blue line). Values are mean ± s.d. (n=3 samples).

1.2. Simulation of the RP effect in EGFP

The differential equations governing the evolution of the concentrations in our final model (Fig. 1f and Supplementary Fig. SD1 with all arrows) are given below (Supplementary Equations SD1-SD5). The photon fluxes F at 470 and 900 nm (in mol.s^{-1}.cm^{-2}) were calculated from the corresponding power densities I (in W/cm^2; Supplementary Equation SD6). The notations used are listed in Supplementary Table SD1.

$$\frac{d[\mathrm{S_0}]}{dt} = -1000 \cdot F_{470} \cdot \varepsilon_{\mathrm{S_0}}^{470} \cdot \ln 10 \cdot [\mathrm{S_0}] + (1 - \Phi_{\mathrm{ISC}}) \cdot k_{\mathrm{S_1}} \cdot [\mathrm{S_1}] + (1 - \Phi_{\mathrm{D}}) \cdot k_{\mathrm{T_1}} \cdot [\mathrm{T_1}] \quad (\mathbf{SD1})$$

$$\frac{d[\mathrm{S_1}]}{dt} = 1000 \cdot F_{470} \cdot \varepsilon_{\mathrm{S_0}}^{470} \cdot \ln 10 \cdot [\mathrm{S_0}] + \Phi_{\mathrm{RISC}} \cdot k_{\mathrm{T_n}} \cdot [\mathrm{T_n}] - k_{\mathrm{S_1}} \cdot [\mathrm{S_1}] \quad (\mathbf{SD2})$$

$$\frac{d[\mathrm{T_1}]}{dt} = \Phi_{\mathrm{ISC}} \cdot k_{\mathrm{S_1}} \cdot [\mathrm{S_1}] - k_{\mathrm{T_1}} \cdot [\mathrm{T_1}] - 1000 \cdot F_{470} \cdot \varepsilon_{\mathrm{T_1}}^{470} \cdot \ln 10 \cdot [\mathrm{T_1}] - 1000 \cdot F_{900} \cdot \varepsilon_{\mathrm{T_1}}^{900} \cdot \ln 10 \cdot [\mathrm{T_1}] + (1 - \Phi_{\mathrm{D_n}} - \Phi_{\mathrm{RISC}}) \cdot k_{\mathrm{T_n}} \cdot [\mathrm{T_n}] \quad (\mathbf{SD3})$$

$$\frac{d[\mathrm{T_n}]}{dt} = 1000 \cdot F_{470} \cdot \varepsilon_{\mathrm{T_1}}^{470} \cdot \ln 10 \cdot [\mathrm{T_1}] + 1000 \cdot F_{900} \cdot \varepsilon_{\mathrm{T_1}}^{900} \cdot \ln 10 \cdot [\mathrm{T_1}] - k_{\mathrm{T_n}} \cdot [\mathrm{T_n}] \quad (\mathbf{SD4})$$

$$\frac{d[\mathrm{D}]}{dt} = \Phi_{\mathrm{D}} \cdot k_{\mathrm{T_1}} \cdot [\mathrm{T_1}] + \Phi_{\mathrm{D_n}} \cdot k_{\mathrm{T_n}} \cdot [\mathrm{T_n}] \quad (\mathbf{SD5})$$

$$F = \frac{I}{E_{photon} \cdot N_{\mathrm{A}}} \quad (\mathbf{SD6})$$

Supplementary Table SD1: Parameters involved in the simulation of the RP effect of EGFP: notations and values used. Process numbers are as defined in Supplementary Fig. SD1. k_X denotes the reciprocal of the lifetime of species X.

Parameter	N. of process	Symbol	Value	Source
Decay rate constant of S_1	2, 3	$k_{\mathrm{S_1}}$	3.85×10^8 [s^{-1}]	Ref. 3
Decay rate constant of T_1	4, 5	$k_{\mathrm{T_1}}$	2.33×10^2 [s^{-1}]	Ref. 1
Decay rate constant of T_n	7, 8, 9	$k_{\mathrm{T_n}}$	1.0×10^{12} [s^{-1}]	Ref. 4
Quantum yield of ISC	3	Φ_{ISC}	0.01	Ref. 1
Quantum yield of RISC	7	Φ_{RISC}	0.03	Data
Quantum yield of T_1 bleaching	4	Φ_{D}	6×10^{-5}	Ref. 5 and data
Quantum yield of T_n bleaching	9	$\Phi_{\mathrm{D_n}}$	4.88×10^{-7}	Data
Extinction coef. of S_0 at 470 nm	1	$\varepsilon_{\mathrm{S_0}}^{470}$	50000 [M^{-1}.cm^{-1}]	Refs. 6,7
Extinction coef. of T_1 at 900 nm[a]	6	$\varepsilon_{\mathrm{T_1}}^{900}$	12000 [M^{-1}.cm^{-1}]	Ref. 1 and data
Extinction coef. of T_1 at 470 nm	10	$\varepsilon_{\mathrm{T_1}}^{470}$	2500 [M^{-1}.cm^{-1}]	Ref. 1 and data

[a] Values of the extinction coefficient of T_1 at other wavelengths used for the simulation of the action spectrum (Supplementary Fig. SD5) were deduced from $\varepsilon_{\mathrm{T_1}}^{900}$ using the T_1 spectrum shape from Ref. 1.

The equations were solved numerically starting from the initial condition $[S_0](t=0) = 2\times10^{-5}$ mol.L^{-1} and $[S_1](t=0) = [S_p](t=0) = [T_1](t=0) = [T_n](t=0) = 0$ in order to obtain the evolution of the concentrations of the different states over time. The fluorescence signal at time t is proportional to the concentration of the bright S_1 state. The simulated RP effect for a given set of 470-nm and 900-nm intensities was therefore calculated as the ratio of the integral of $[S_1](t)$ in presence of NIR light to its integral in absence of NIR light. We fixed the values of the different photophysical parameters (Supplementary Table SD1) either based on the available literature or in such a way as to minimize the difference between the simulated and measured RP effect at the different 470-nm and 900-nm light intensities (Fig. 1c,d).

The simulated concentration profiles of S_0, S_1, T_1, T_n and D under 470-nm illumination alone (32 W/cm^2) or combined with saturating 900-nm co-illumination (2 kW/cm^2) are shown in Supplementary Fig. SD4. At the onset of 470-nm illumination, a small population is transferred in a few ns from S_0 to S_1 (panel a), leading to the fluorescence signal. In the absence of NIR light, S_1 and S_0 populations which are in rapid equilibrium then partially convert to T_1 on the ms timescale (b and e, dotted lines), due to intersystem crossing from S_1 to T_1. This results in a significant T_1 population and a new equilibrium between S_0, S_1 and T_1. These three populations finally decay on the timescale of a few minutes due to conversion of T_1 to D (c, d and f, dotted lines). By promoting RISC, NIR light drastically reduces T_1 population (e and f, solid orange line) in favor of S_0 (solid blue line). S_1 and T_n populations are also enhanced (b, c, g and h, solid cyan and red lines). The increase in T_n population makes photobleaching from T_n dominant. With $[T_n]/[T_1] = 4.1 \times 10^{-7}$ and rate constants $k_{Dn} = \Phi_{Dn} \times k_{Tn} = 4.9 \times 10^5$ s^{-1} and $k_D = \Phi_D \times k_{T1} = 1.4 \times 10^{-2}$ s^{-1} (Supplementary Table SD1), the ratio of the photobleaching rates from T_n and T_1 is in fact $v_{Dn}/v_D = k_{Dn}/k_D \times [T_n]/[T_1] \approx 14$. The photobleaching rate from T_n in these conditions is however smaller than the photobleaching rate from T_1 in the absence of NIR light (Supplementary Fig. SD4d, green lines) due to the much smaller population of T_n than T_1.

Our model enabled us to satisfactorily simulate the experimental dependence of the RP effect on I_{470} and I_{900} (Fig. 1c,d). We also used it to generate a contour plot of the RP effect as a function of the two light intensities in the range of 1-10^4 W/cm^2 (Fig. 1g). This plot shows that the effect is at maximum ($\geq$ 3-fold increase of the time-integrated emission of EGFP) in the rectangle defined by $I_{470} \leq 100$ W/cm^2 and $I_{900} \geq 500$ W/cm^2. Finally, we simulated the NIR wavelength dependence of the RP effect (action spectrum; Supplementary Fig. SD5) using values of the molar extinction coefficient of T_1 at the different wavelengths deduced from its value at 900 nm (Supplementary Table SD1) and the T_1 spectrum shape from Ref. 1. The simulation appropriately predicts the correspondence with T_1 spectrum at low NIR intensity as well as the observed broadening at high intensity. The latter comes from the saturation of the RP effect at high NIR intensity, a regime in which the RP effect depends less on the absorbed light intensity, and therefore on the variation of the extinction coefficient with the wavelength.

Although it predicts the dependencies of the RP effect on visible and NIR intensities and NIR wavelength, our model does not fit the experimental photobleaching kinetics well (Supplementary Fig. SD6). Indeed, a satisfactory fit of the data requires three exponential components (Supplementary Fig. SD7), while the simulated photobleaching kinetics contain only one (Supplementary Fig. SD8) – neglecting fast components due to S_0 to S_1 and S_1 to T_1 conversions (Supplementary Fig. SD4, a and b). This suggests that the photophysics of EGFP involves more states than assumed in our model. In agreement with previous reports,[8-10] we observed a small reversible photobleaching component in EGFP (Supplementary Fig. SD9) indicating the formation of low-yield, long-lived dark states under illumination. These states could correspond to isomerized, hydrated or radical forms.[1,11-13] While such states could lead to additional components in the fluorescence decay, they are not expected to be sensitive to NIR light. Moreover, the action spectrum of the RP effect (Fig. 1e) indicates clearly that it originates from re-excitation of the triplet state.

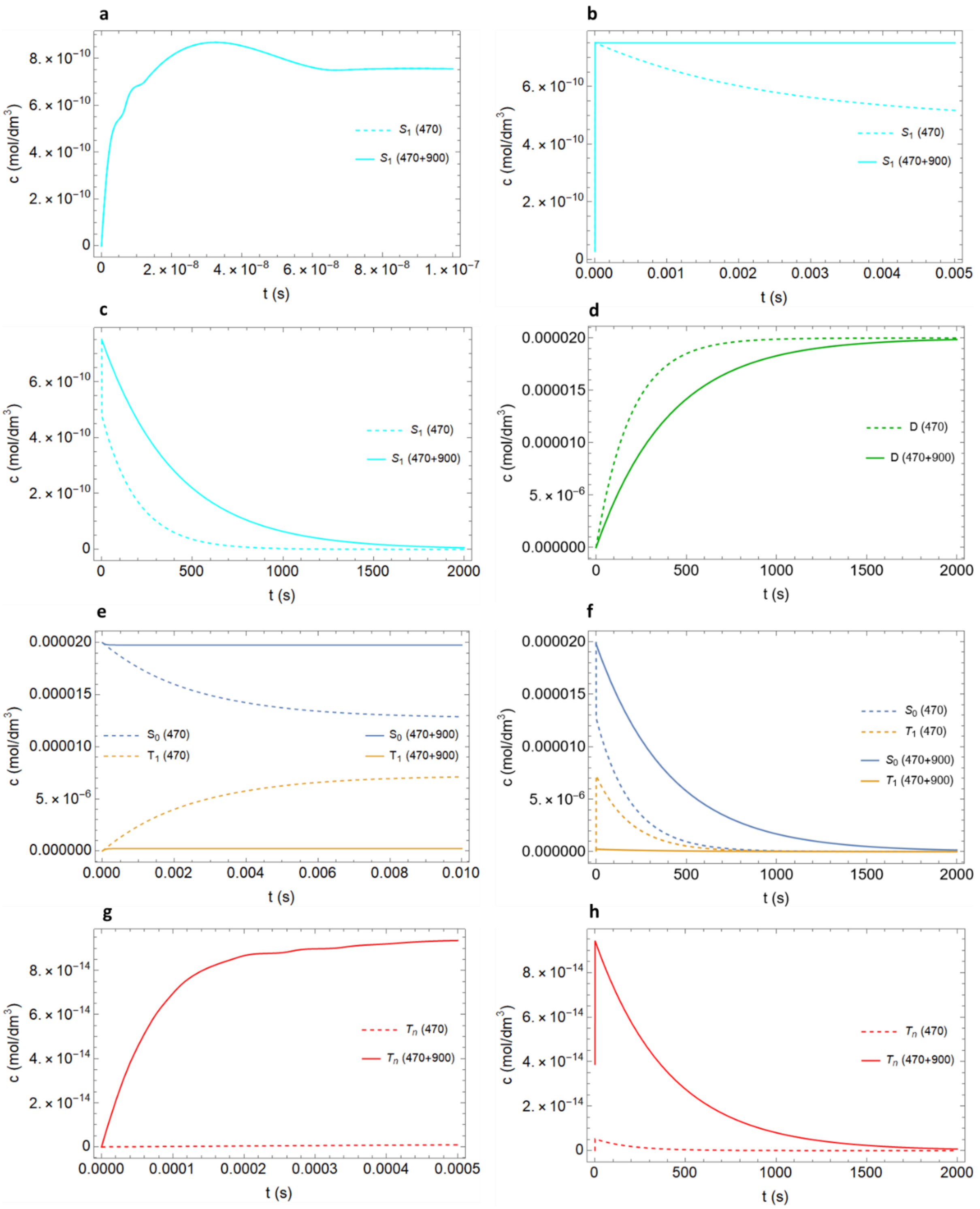

Supplementary Fig. SD4: Simulated concentration changes of S_1 (a, b, c), D (d), S_0 and T_1 (e, f) and T_n (g, h) under continuous illumination at 470 nm alone (32 W/cm^2, dashed lines) or combined with a saturating co-illumination at 900 nm (2 kW/cm^2, solid lines), shown on different timescales. Note that S_1 nanosecond rise due to 470-nm illumination is independent of the presence of NIR light. S_1 concentration profiles with and without NIR are therefore superimposed on this timescale (panel a).

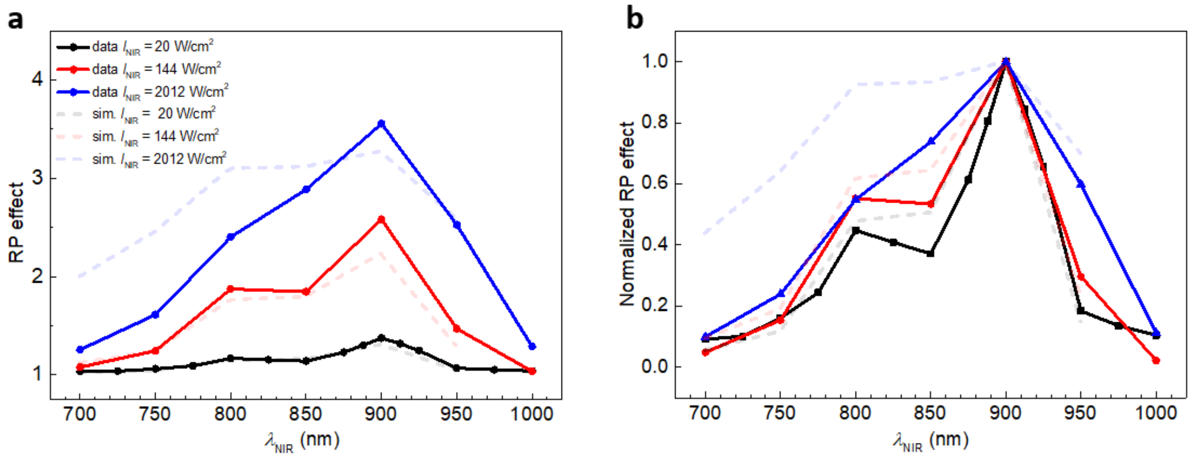

Supplementary Fig. SD5: a, Experimental (solid lines) and simulated (dashed lines) RP effect dependence on the wavelength of NIR light for different NIR intensities and a fixed 470-nm intensity of 32 W/cm^2. **b,** Same, normalized to the 0 to 1 range to highlight the broadening at high NIR intensity.

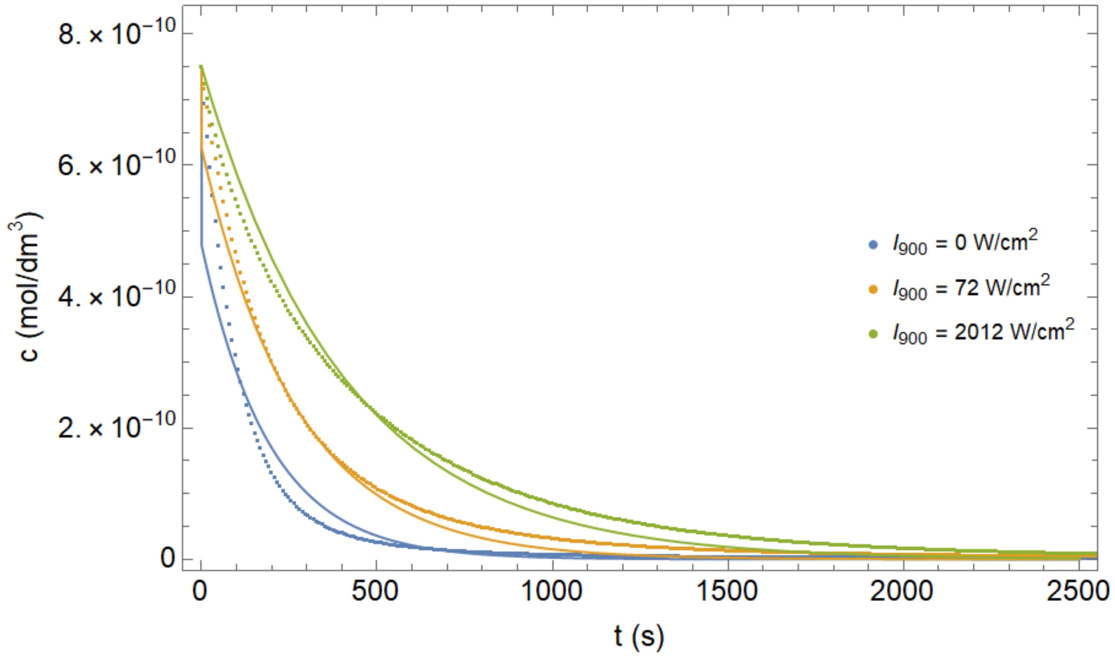

Supplementary Fig. SD6: Comparison of the simulated concentration profiles of S_1 (solid lines) with the experimental photobleaching kinetics of purified EGFP in PAA gel (dotted lines) under 470-nm illumination at 32 W/cm^2 and 900-nm co-illumination at different intensities. The experimental data were normalized to S_1 molar concentration.

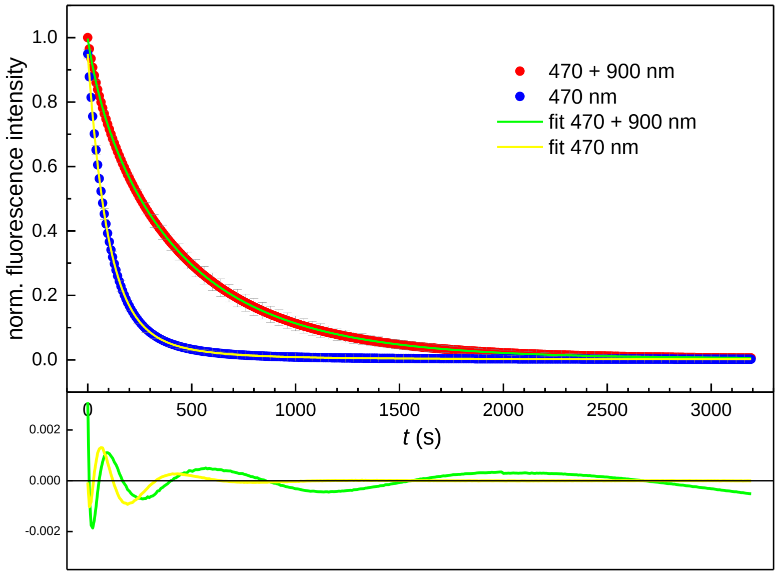

Supplementary Fig. SD7. Three-exponential fits of the photobleaching kinetics of purified EGFP in PAA gel under continuous illumination at 470 nm (32 W/cm^2; blue circles, yellow line) alone or combined with 900 nm (2012 W/cm^2; red circles, green line. Data are mean ± s.d. (n=4 different samples). The bottom panel shows the residuals of the fits. The obtained lifetimes are 93 s (84 %), 267 s (15 %) and 1103 s (1 %) for 470-nm illumination and 49 s (9 %), 243 s (27 %) and 559 s (64 %) for dual illumination, where percentages indicate the relative weight of each component.

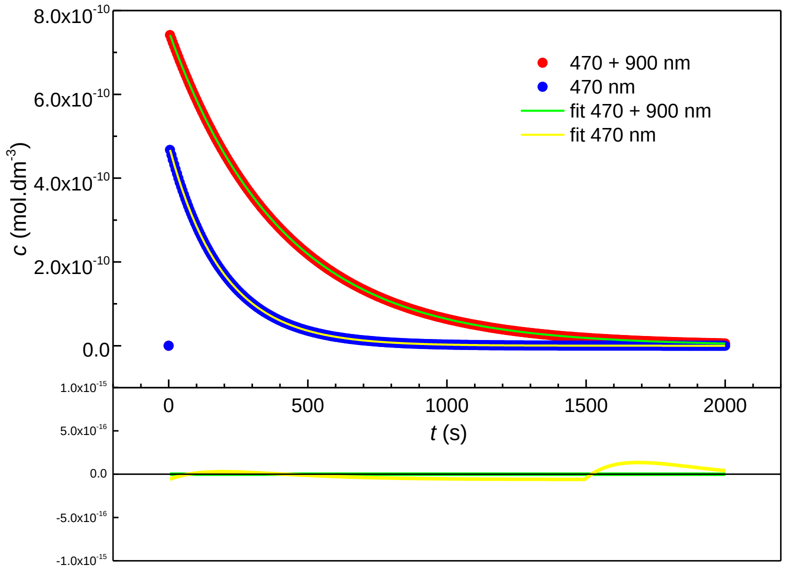

Supplementary Fig. SD8. Mono-exponential fits of the simulated concentration profiles of S_1 under illumination at 470 nm (32 W/cm^2; blue and yellow lines) alone or combined with 900-nm co-illumination (2012 W/cm^2; red and green lines). The bottom panel shows the residuals of the fits. The obtained lifetime is 193 s for 470-nm illumination and 406 s for dual illumination. Fast components due to S_0 to S_1 and S_1 to T_1 conversions were neglected.

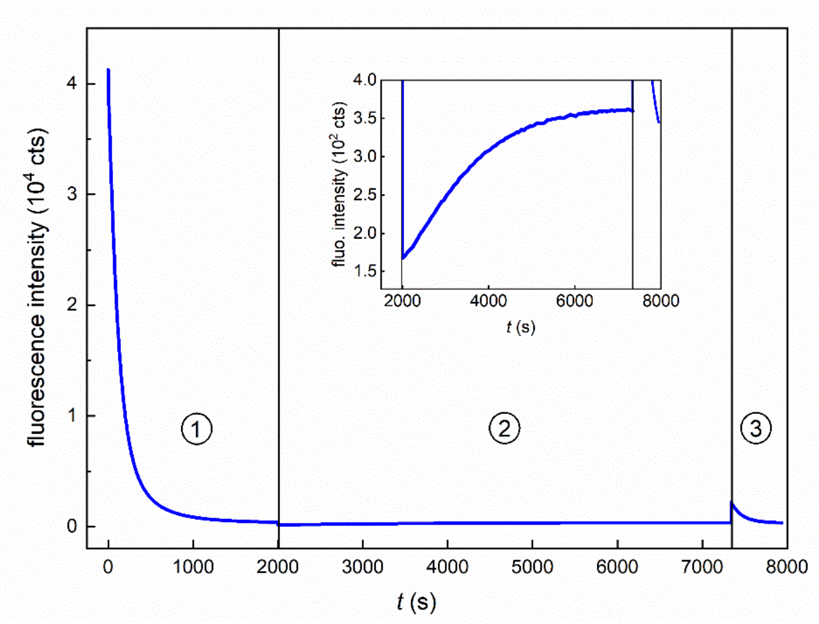

Supplementary Fig. SD9. Reversible photobleaching of EGFP. A sample of purified EGFP immobilized in a PAA gel was first continuously illuminated with 32 W/cm^2 of 470-nm light until fully photobleached (1). In a 2nd phase (2), the intensity was reduced to 3.2 W/cm^2 which led to a slight recovery of the fluorescence on the time scale of 1000 s, as highlighted in the inset. In a 3rd phase (3), the intensity was increased back to 32 W/cm^2. The partial fluorescence recovery during the 2nd phase as well as the higher level of fluorescence at the beginning of the 3rd phase than at the end of the 1st phase show the presence of a small component of reversible photobleaching in EGFP. Note that the instantaneous changes in fluorescence intensity at the boundaries between the different phases simply reflect the changes in excitation intensity.

1.3. Sensitivity of the RP effect to the fluorophore's photophysical parameters

We show in Fig. 1h that beyond EGFP, the RP effect can be obtained in a wide range of FPs, with however variable amplitudes. In addition, RISC is not specific to FPs but has been demonstrated in a number of organic dyes such as cyanines[14] or xanthenes,[15] suggesting that the RP effect could also occur in small fluorophores. Interestingly, large increases in instantaneous fluorescence assigned to RISC have been reported for several small fluorophores subjected to dual illuminations at extremely high intensities (0.1-10 MW/cm^2).[2]

In order to understand, or predict, the variations of the RP effect from one fluorophore to another, we examined its sensitivity to the photophysical parameters of fluorophores (molar absorption coefficient of T_1, quantum yield of RISC, quantum yields of bleaching from T_1 and from T_n, quantum yield and lifetime of T_1; Supplementary Fig. SD10-SD12), in the framework of the model of Fig. 1f. The wavelength used to excite the ground-state is noted here λ_1 (with intensity I_1) and that used to excite the triplet state λ_2 (with intensity I_2). Each figure panel illustrates the sensitivity of the RP effect to one parameter, with all other parameters set to the values in Supplementary Table SD1.

Changing the value of $\varepsilon_{T_1}(\lambda_1)$ impacts the dependence of the RP effect on I_1 (Supplementary Fig. SD10a), where with a higher $\varepsilon_{T_1}(\lambda_1)$ value the RP effect is observable only at a lower range of I_1. Logically, when $\varepsilon_{T_1}(\lambda_1) = 0$ the RP effect is independent on I_1. Similarly, the value of $\varepsilon_{T_1}(\lambda_2)$ impacts the dependence of the RP effect on I_2 (Supplementary Fig. SD10b). The higher $\varepsilon_{T_1}(\lambda_2)$, the less I_2 is required to observe the RP effect.

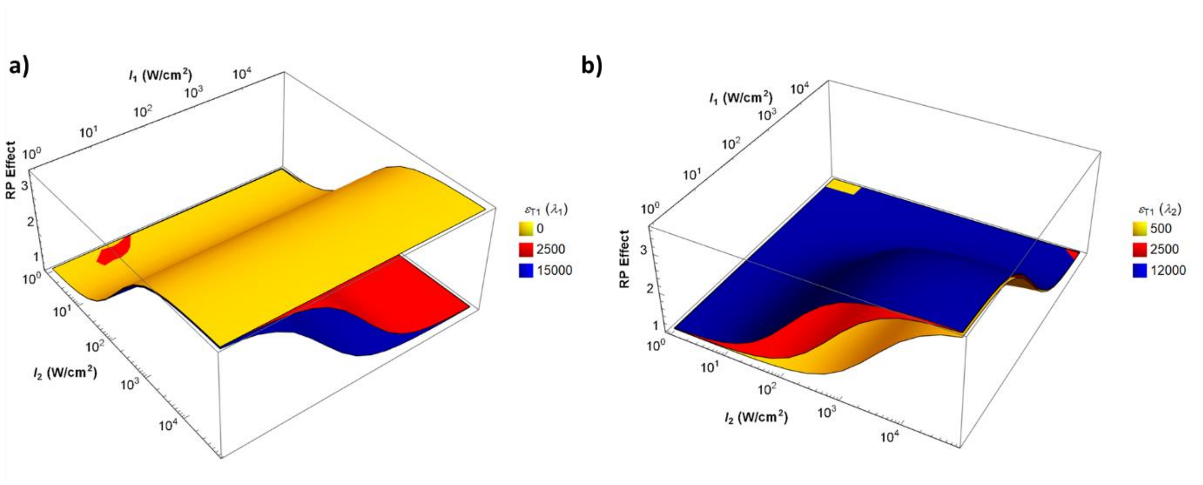

Supplementary Fig. SD10: Simulation of the RP effect for different values of a) the molar absorption coefficient of the triplet state at λ_1 ($\varepsilon_{T_1}(\lambda_1)$) and b) the molar absorption coefficient of the triplet state at λ_2 ($\varepsilon_{T_1}(\lambda_2)$). All other photophysical parameters were set to the values in Supplementary Table SD1.

The quantum yield of ISC (Φ_{ISC}) influences the magnitude of the effect: the higher the value of Φ_{ISC}, the lower the RP effect (Supplementary Fig. SD11a). The triplet lifetime (τ_{T_1}) changes the dependency in both I_1 and I_2 (Supplementary Fig. SD11b). For fluorophores with short triplet lifetimes in the microsecond range (such as small organic fluorophores) the RP effect is expected to be present over a broader range of I_1 intensities (from 1 W/cm^2 up to ~10 kW/cm^2), but to require very high I_2 intensities (> 100 kW/cm^2). Conversely, for fluorophores with long triplet lifetimes in the tens of milliseconds range, the effect is expected to be present only at very low I_1 intensity (< 10 W/cm^2) and to require lower I_2 intensity (≥ 100 W/cm^2). Indeed, the longer the triplet lifetime, the more effective the interaction with the illumination beams. This favors RISC induced at both λ_1 and λ_2. The threshold value of I_1 beyond which the effect decreases is then shifted towards low intensities, as is the threshold value of I_2 above which the effect is maximum. The fact that we observe a significant RP effect for most green and yellow FPs in the illumination conditions optimized for EGFP (τ_{T_1} = 4.3 ms[1]) suggests that their triplet lifetimes are in the same millisecond range.

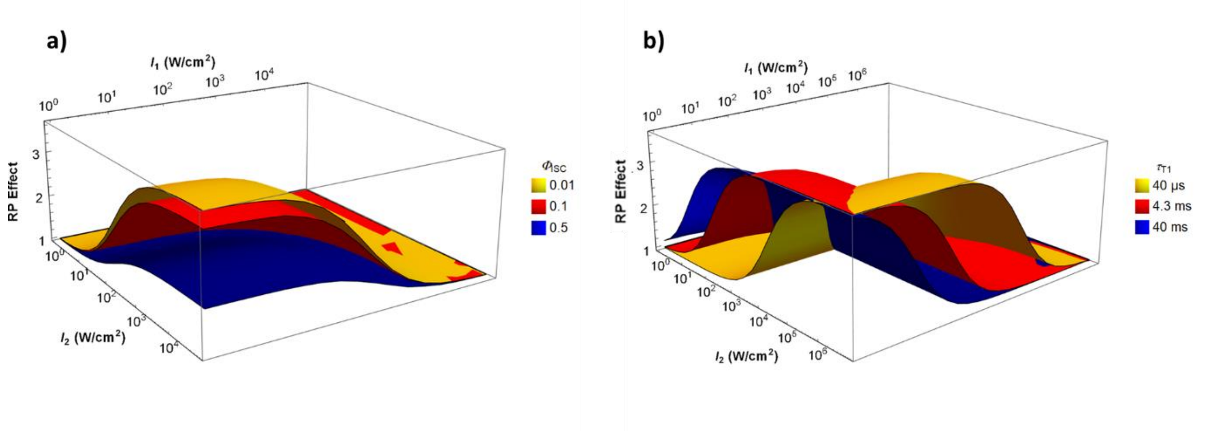

Supplementary Fig. SD11: Simulation of the RP effect for different values of a) the quantum yield of ISC (Φ_{ISC}) and b) the lifetime of T_1 (τ_{T_1}). All other photophysical parameters were set to the values in Supplementary Table SD1.

The values of the quantum yield of RISC (Φ_{RISC}), bleaching from T_1 (Φ_D) and bleaching from T_n (Φ_{D_n}) affect the magnitude of the RP effect but not its dependence on the intensities at λ_1 and λ_2 (Supplementary Fig. SD12). The higher the values of Φ_{RISC} and Φ_D, the greater the RP effect. Conversely, increasing the value of Φ_{D_n} decreases the effect. Some combinations of values of these three parameters can lead to a RP effect < 1, that is an acceleration of the photobleaching under dual illumination. This could in particular explain the increase in photobleaching observed for bacteria labelled with mCherry or mRFP1 and grown on PBS-agarose pads (Supplementary Fig. 3c-d). In order to achieve a RP effect > 1, Φ_{D_n} must be at least 4-5 orders of magnitude lower than Φ_{RISC} and 2 orders of magnitude lower than Φ_D.

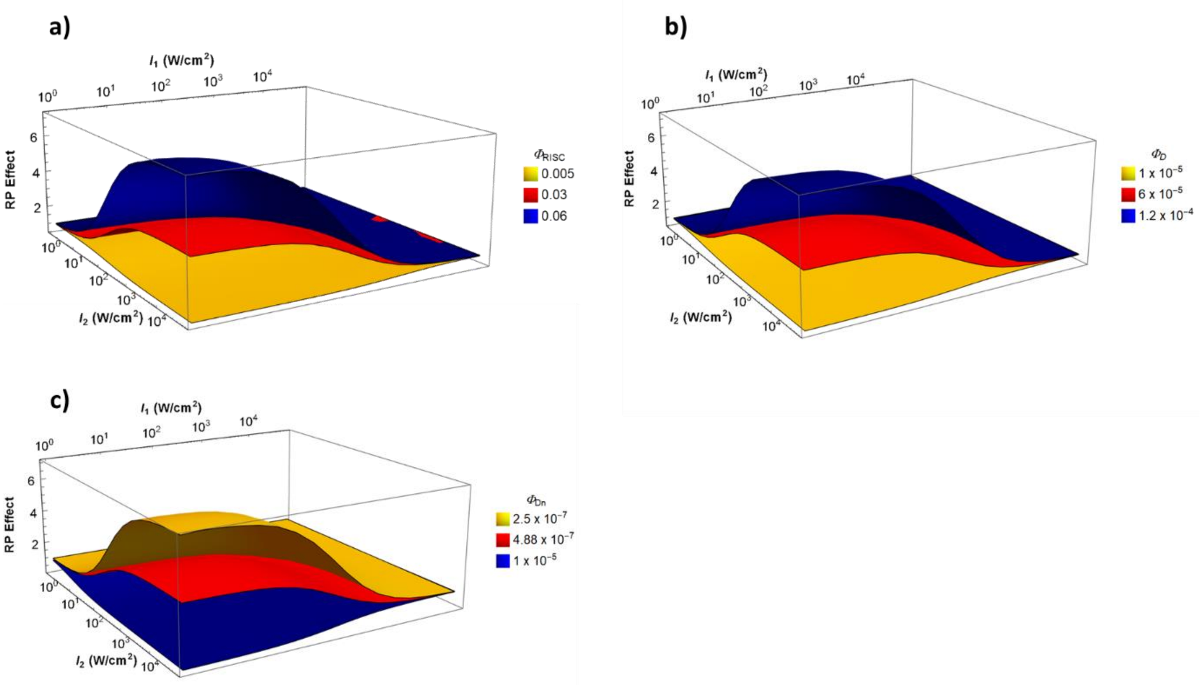

Supplementary Fig. SD12: Simulation of the RP effect for different values of a) the quantum yield of RISC (Φ_{RISC}), b) the quantum yield of bleaching from T_1 (Φ_D) and c) the quantum yield of bleaching from T_n (Φ_{D_n}). All other photophysical parameters were set to the values in Supplementary Table SD1.

In conclusion, the variations in amplitude of the RP effect among green and yellow FPs (Fig. 1h) are most likely due to slight variations in several of the photophysical parameters discussed above.

As far as small fluorophores are concerned, we expect the RP effect to take place in a higher intensity regime than for FPs (more precisely, over a wider range of I_1 intensities and for higher I_2 intensities) because their triplet lifetimes are ~1000 times shorter (microsecond scale). For instance, for fluorescein whose triplet lifetime is 33 µs,[16] we expect an onset of the RP effect for I_2 intensities of a few kW/cm^2, and a maximum effect from several 100 kW/cm^2 (Supplementary Fig. SD13a). We checked this prediction experimentally by exciting fluorescein at 470 nm (35 W/cm^2) and re-exciting its triplet in its absorption tail at 700 nm[17] to induce RISC. In agreement with our prediction, we observed an increase in time-integrated emission with 2.5 kW/cm^2 of 700-nm light (maximum intensity achievable with our setup) while 250 W/cm^2 proved insufficient to obtain an effect (Supplementary Fig. SD13b). It is

interesting to note that unlike FPs for which the time-integrated emission increases due to a slowing down of photobleaching, here the increase is mainly due to an instantaneous increase in fluorescence at *t*=0. This observation is in agreement with the work of Ringemann et al. who reported large increases in instantaneous fluorescence for several small fluorophores under dual illumination at very high intensities (0.1-10 MW/cm^2).[2]

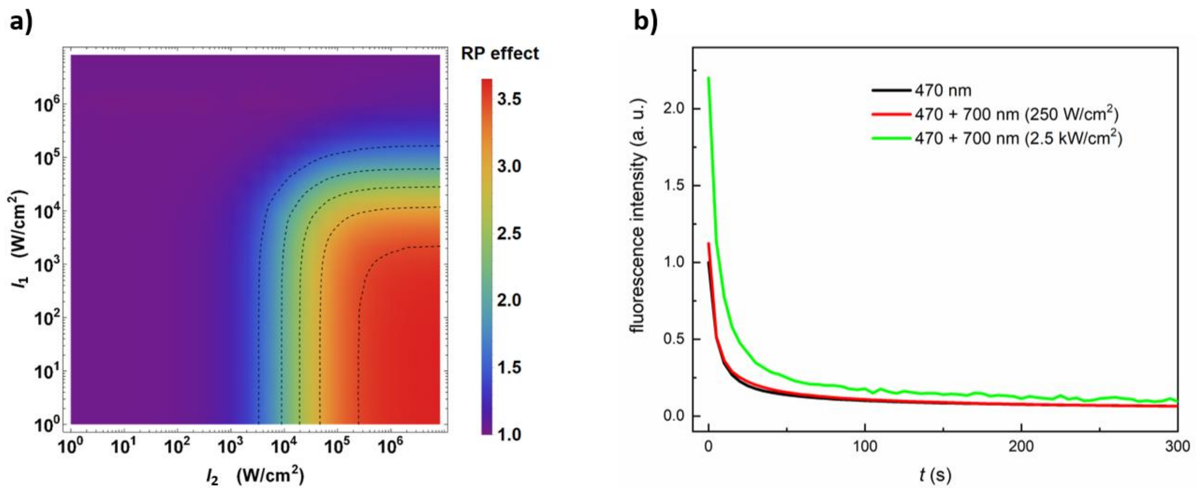

Supplementary Fig. SD13: RP effect in fluorescein. a) Simulated intensity-dependence of the RP effect for a fluorophore with a triplet lifetime of 33 µs. All other photophysical parameters were kept to their EGFP values listed in Supplementary Table SD1. b) Fluorescence photobleaching curves of fluorescein under continuous illumination at 470 nm (35 W/cm^2) alone or combined with 700 nm. Fluorescein was immobilized in a polyvinyl alcohol (PVA) film obtained by drying a drop of a 20-µM dye solution containing 1 wt% PVA.[18]

2. Supplementary notes

Supplementary Note 1

Contribution of endogenous photosensitizers to phototoxicity:

Phototoxicity occurs when molecules in biological samples absorb the excitation light and interact in their triplet excited state with oxygen to produce ROS.[19] These molecules are called photosensitizers. In a labelled sample, phototoxicity can arise not only from the fluorophore, but also from endogenous photosensitizers, among which flavins and porphyrins.[20,21] Due to the specific absorption spectra of endogenous photosensitizers, the relative contributions of the fluorophore and the endogenous photosensitizers to phototoxicity in a given sample depend on the excitation wavelength.[22] At a given excitation wavelength, these contributions may also depend on the cell type because the nature and amounts of endogenous photosensitizers vary from one cell type to another.[22,23] Finally, they depend on the nature of the fluorophore and its efficiency to produce ROS under excitation, which could be related to its photobleaching rate[24,25] (although different fluorophores may have different photobleaching mechanisms, leading to different amounts of ROS[26,27]).

In the present work, NIR co-illumination is only effective against phototoxicity due to FPs. Thus, the fact that it is not effective for EGFP-labelled bacteria excited at 490 nm (Supplementary Fig. 11a) suggests that endogenous phototoxicity dominates in these conditions. Conversely, the fact that NIR co-illumination completely suppresses phototoxicity for YPet-labelled bacteria excited at 517 nm (Fig. 2h-i) suggests that endogenous phototoxicity is negligible in that case. Although we cannot rule out that YPet is a greater producer of ROS than EGFP (it photobleaches faster, see Supplementary Fig. 1), our control experiments on unlabelled bacteria suggest that endogenous phototoxicity is significant at 490 nm and negligible at 517 nm (Supplementary Fig. 11b-c). This difference could be due to the presence of flavins, whose absorption is substantial between 450 and 500 nm but almost zero at 517 nm.[28]

In this context, it may seem surprising that NIR co-illumination reduces phototoxicity for GFP-labelled neutrophils excited at 470 nm (Fig. 2f-g). It is however well documented that mammalian cells are less sensitive to blue light than bacteria,[29] likely because they have a different content in endogenous photosensitizers.

Supplementary Note 2

Deep neural network (DNN) for neutrophil segmentation:

The DNN architecture was based on a 5-layers UNet,[30] with each layer containing convolutions with 32, 32, 64, 128, 256 filters, respectively and without the skip connection at the first layer. The network was trained for 1000 epochs of 200 steps with a batch size of 64, using Adam optimizer and a learning rate starting from $2e^{-4}$ which was reduced by a factor 2 on plateau of 20 epochs. The categorical cross-entropy loss was balanced by class frequency. The training set consisted of 9 annotated images of the control condition, which had the lowest SNR among all conditions. The annotations where made using the BACMMAN software in approximately 30 minutes. Importantly, the loss was only computed on annotated areas, which allowed for partial annotation of images, and selection of difficult examples within the images. Images where randomly zoomed in the range [0.8, 1.2], cropped to a size of 512×512, rotated, and transformed using elastic deformation and Gaussian blur. Intensity was randomly scaled and Gaussian noise was added to improve robustness to the large variability in intensity and signal-to-noise ratio (SNR) among conditions and within images.

3. Supplementary figures

Supplementary Fig. 1.

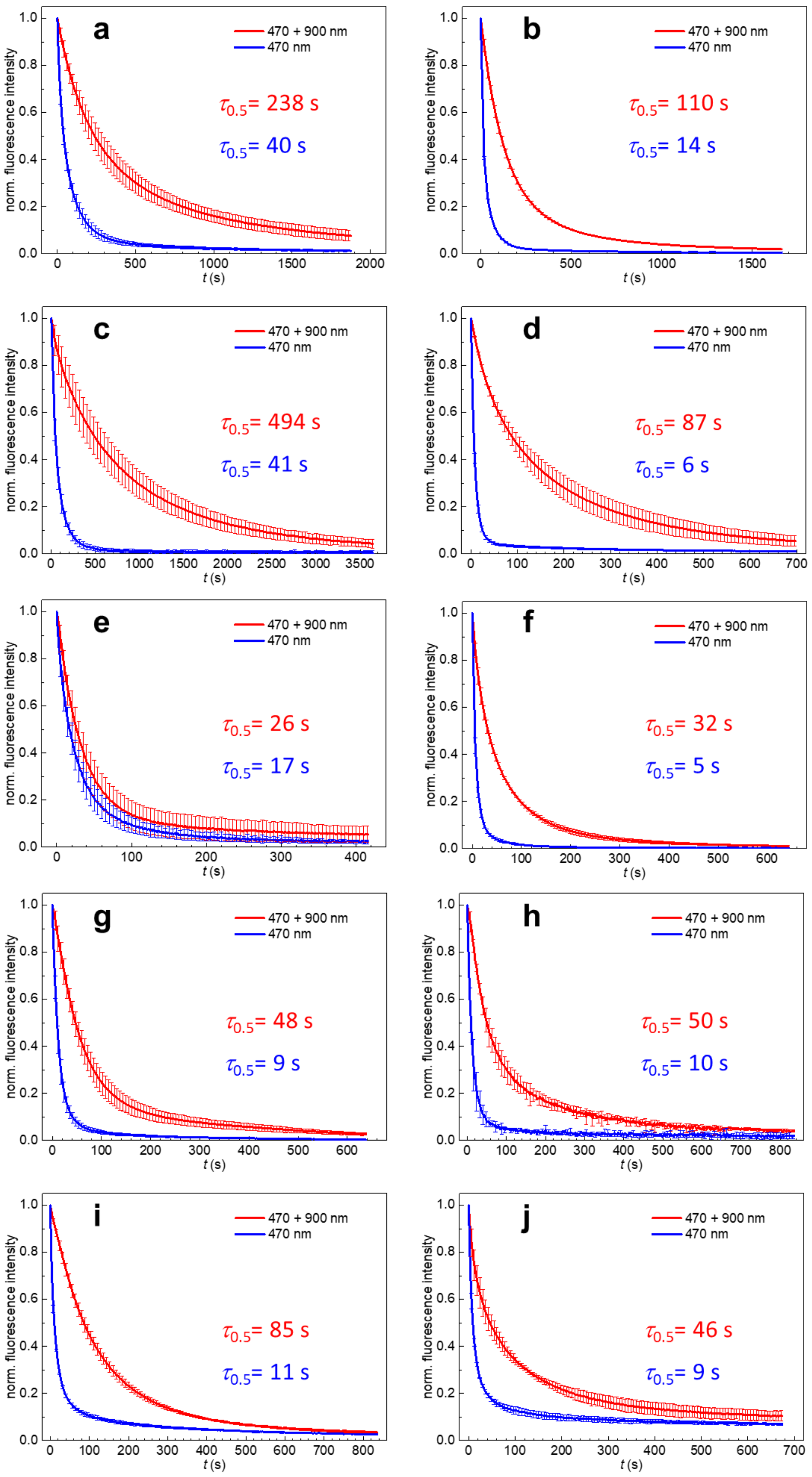

Photobleaching kinetics of different green and yellow FPs expressed in *E. coli*:
a, EGFP. **b,** sfGFP. **c,** acGFP. **d,** mClover. **e,** mWasabi. **f,** emGFP. **g,** Venus. **h,** EYFP. **i,** YPet. **j,** Citrine. The bacteria were deposited on a PBS-agarose pad and continuously illuminated at 470 nm (32 W/cm^2, blue) alone or combined with 900 nm (2 kW/cm^2, red). The values are mean ± s.d. (n=3 samples). The fluorescence half-lives ($\tau_{0.5}$) of the different FPs in the presence and absence of NIR light are specified in the corresponding panels.

Supplementary Fig. 2

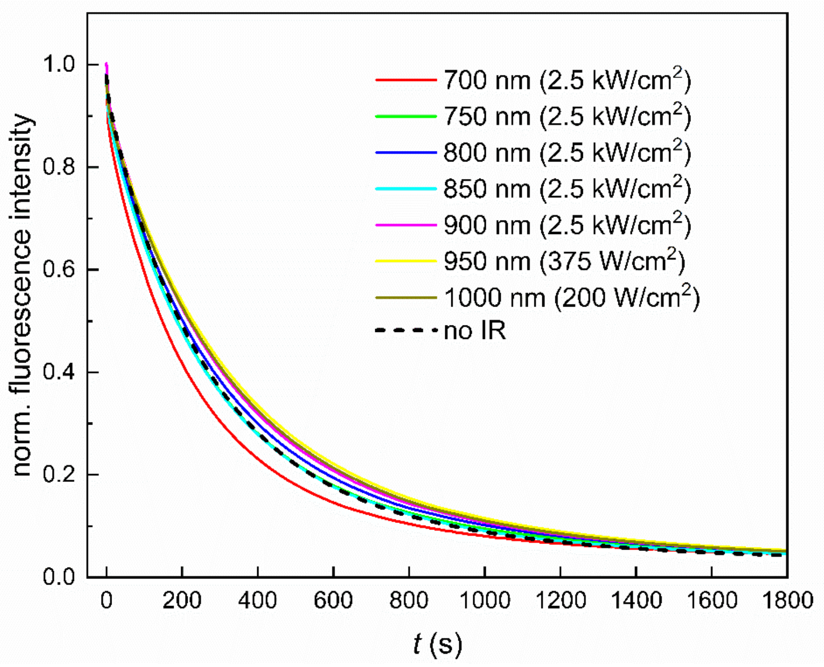

Absence of RP effect for ECFP between 700 and 1000 nm:
Purified ECFP was immobilized in a PAA gel and continuously illuminated at 470 nm alone (35 W/cm^2) or combined with NIR light of different wavelengths. We did not observe any significant effect of NIR co-illumination in the range of wavelengths and intensities accessible with our setup.

Supplementary Fig. 3.

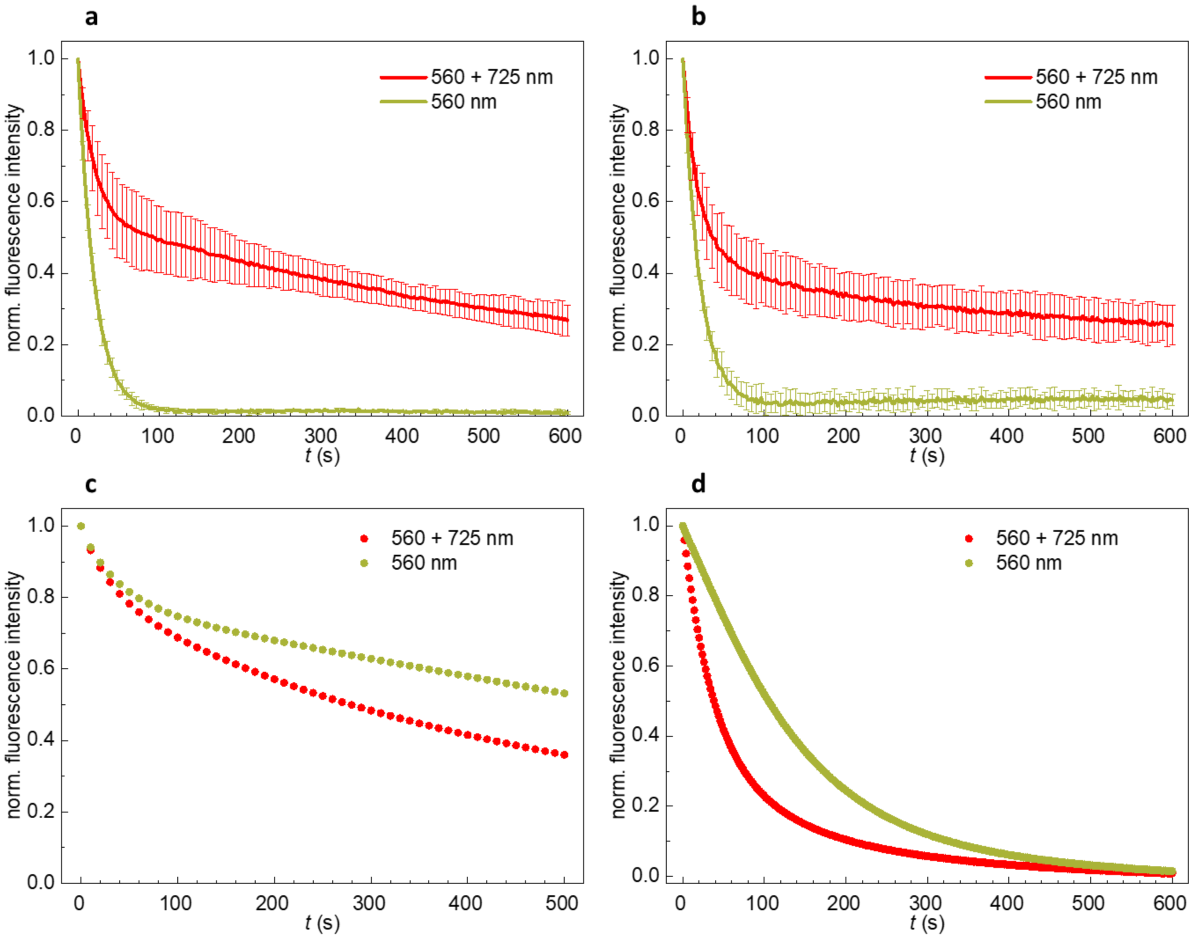

Photobleaching kinetics of red FPs expressed in *E. coli*:
a, mCherry. **b,** mRFP1. The bacteria were deposited on a **LB-agarose** pad and continuously illuminated at 560 nm (5.8 W/cm^2, green-yellow) alone or combined with 725 nm (248 W/cm^2, red). The values are mean ± s.d. (n=1 sample, 3 measurements).
c, mCherry. **d,** mRFP1. The bacteria were deposited on a **PBS-agarose** pad and continuously illuminated at 560 nm (5.8 W/cm^2, green-yellow) alone or combined 725 nm (248 W/cm^2, red).

In the case of red FPs we observed a reduction in photobleaching upon NIR co-illumination for labelled bacteria growing on a LB-agarose pad (panels a and b above). The maximum effect was observed for a NIR wavelength of ~725 nm, and a strong effect was also obtained with 800-nm light. Photobleaching was however increased by NIR light when the bacteria were deposited on a PBS-agarose pad (panels c and d). Such increased photobleaching was also observed for purified mCherry in a polyacrylamide gel and for mCherry expressed in eukaryotic cells (data not shown). Further studies are in progress to understand these environment effects. Note that such environment effects were not observed in green and yellow FPs, for which the photobleaching reduction occurred in all environments tested (purified proteins in PAA, bacteria on LB or PBS-agarose pads and mammalian cells).

Supplementary Fig. 4.

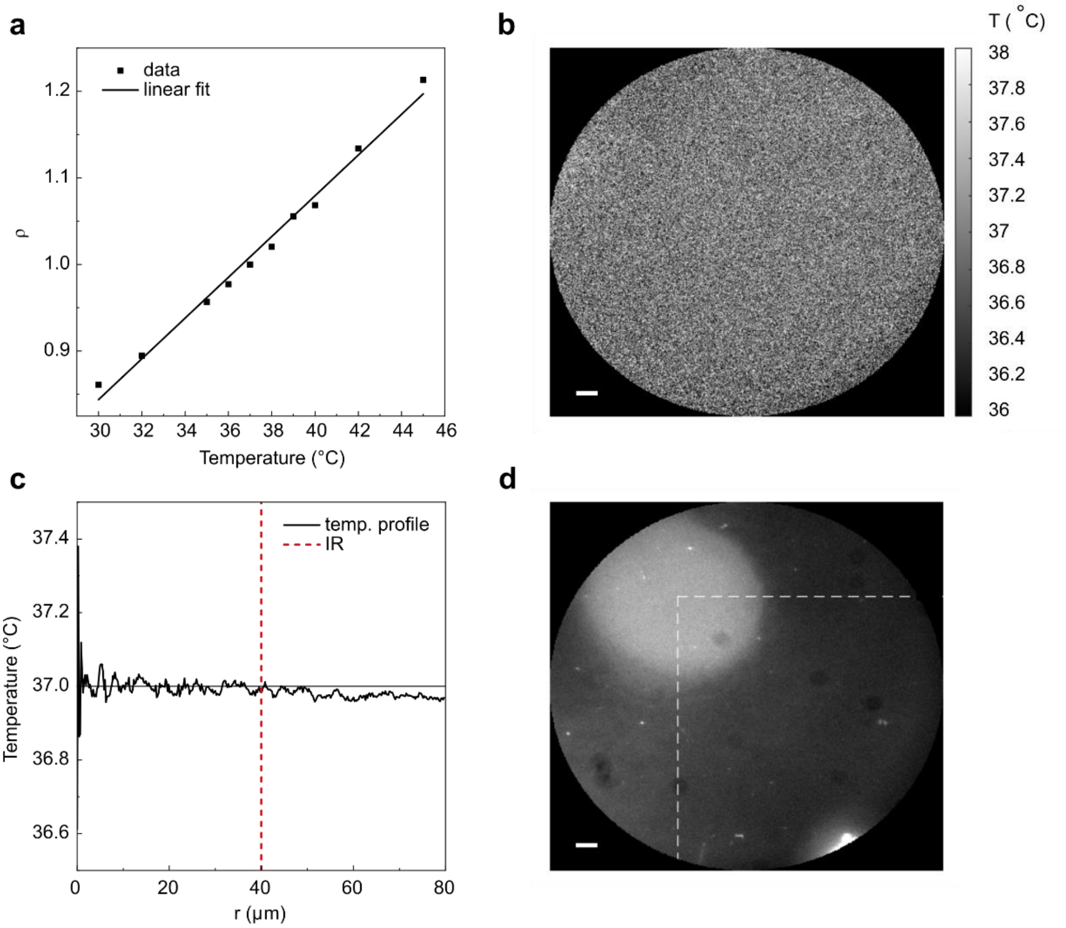

Sample temperature in the presence of the NIR beam:

To check that the NIR beam does not induce any significant heating of the samples due to its absorption by water, we performed temperature measurements using a DNA-based ratiometric fluorescent probe.[31] A rise in temperature leads to a change in the conformation of the probe that decreases the FRET efficiency between two fluorophores (Fluorescein and Texas Red), hence increasing the ratio of the probe's fluorescence intensity in the green to its fluorescence intensity in the red. The transition temperature can be adjusted by modulating the concentration of $MgCl_2$ in the solution.

a, Calibration curve. Temperature dependence of the ratio ρ of probe intensities in the green and red channels, normalized to its value at 37°C, for a $MgCl_2$ concentration of 2 mM.

b, Temperature map of a 2-mm thick probe solution (typical medium height in mammalian cell samples) thermostated at 37 ± 0.2 °C and continuously illuminated at 470 nm (0.2 W/cm^2, whole field) and 900 nm (1.7 kW/cm^2, 80-µm diameter spot). Weaker illuminated areas at the edges have been masked. Scale bar: 10 µm. Representative of n=3 replicate experiments.

c, Radial temperature profile as a function of distance r from the center of the NIR beam, obtained from the map shown in b. The dotted red line indicates the edge of the NIR beam.

d, Fluorescence image of an EGFP-PAA gel sample after simultaneous illumination at 470 nm and 900 nm under the same conditions as in b, showing the position of the NIR beam as a bright disk. The dotted white lines indicate the angular sector over which the temperature data were averaged to obtain the radial profile shown in c. The bright spots are due to fluorescent beads embedded in the gel to facilitate focusing. Scale bar: 10 µm. Representative of n=3 replicate experiments.

The measurement was repeated three times and the temperature rise inside the NIR beam never exceeded 0.1 °C. Note that this measurement was carried out under continuous illumination by the NIR beam, and that the temperature rise would be even lower for discontinuous illumination as used in time-lapse microscopy. We therefore expect no difficulties for wide-field microscopy applications with medium to high magnification (40x, 60x and 100x objectives). The temperature issue may however need to be reassessed if the total incident power of the NIR beam were to increase, for example to cover a field of view >200 µm with a NIR power density >1 kW/cm^2.

Supplementary Fig. 5.

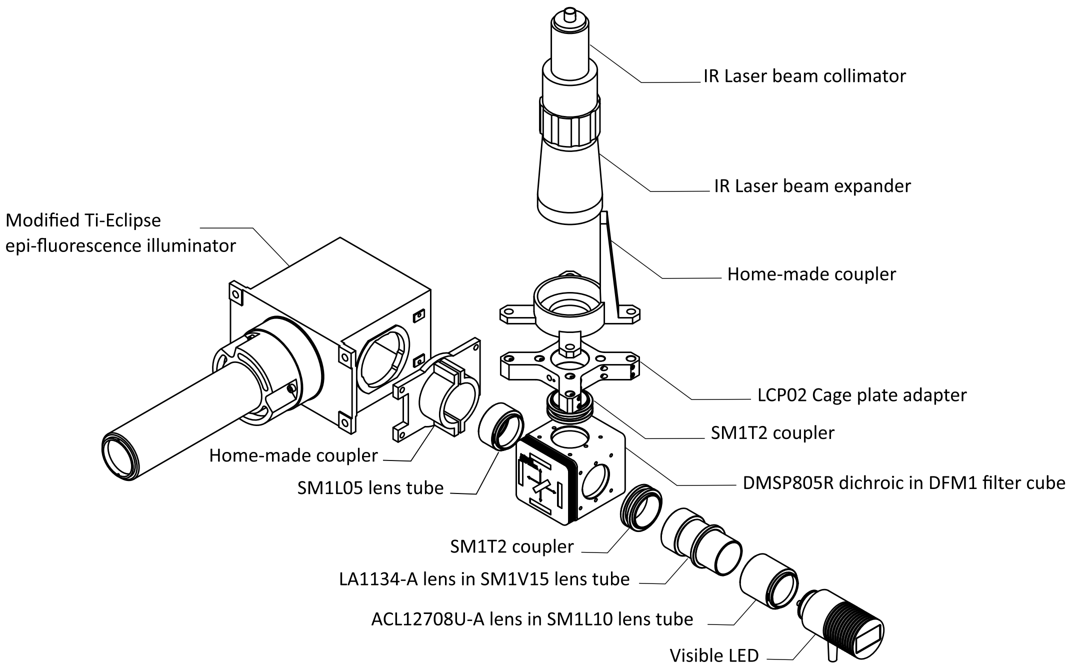

Modification of the illuminator of the Nikon Eclipse Ti microscope to achieve dual visible and NIR illumination over the same field of view:

The epi-fluorescence illuminator of the Nikon microscope was modified as follows. The original illuminator consists of two parts, the main part which is connected to the light source, and the connection part which is inserted into the microscope body. We removed the main part and kept only the connection part, which contains a mirror and a converging lens. The NIR light source was a 1-W 885-nm laser diode (RLTMDL-885-1W with PSU-LED power supply, Roithner) and the visible light source was a LED (mounted M490L4 or M530L4 LED, Thorlabs). Visible and NIR light were combined using a shortpass dichroic mirror with cutoff at 805 nm (DMSP805R, Thorlabs) inserted into a kinematic fluorescence filter cube (DFM1, Thorlabs). Before entering this cube, visible light from the LED was collimated using two lenses (ACL12708U-A and LA1134-A, Thorlabs). The aspheric condenser lens ACL12708U-A was mounted into a SM1L10 lens tube with an AD1T adapter and connected to the LED. On the other side, the lens tube was connected to a SM1V15 lens tube containing the plano-convex lens LA1134-A. This lens tube was connected on the other side to the DFM1 filter cube using a SM1 coupler (SM1T2, Thorlabs). IR light from the laser entered a fiber (RLTMXL FC-400, Roithner) and was then collimated using a collimator (RLTMXL FOC-01A, Roithner) and expanded using a beam expander (RLTMXL LBEX5, Roithner). This beam expander was connected to the DFM1 filter cube with a SM1 coupler (SM1T2, Thorlabs), a cage plate adapter (LCP02, Thorlabs) and a home-made coupler that we fabricated using 3D printing. The DFM1 cube was connected to the conserved part of the Nikon illuminator with another home-made coupler combined with a lens tube (SM1L05, Thorlabs). The total cost of implementing NIR co-illumination was approximately $6,000.

Supplementary Fig. 6.

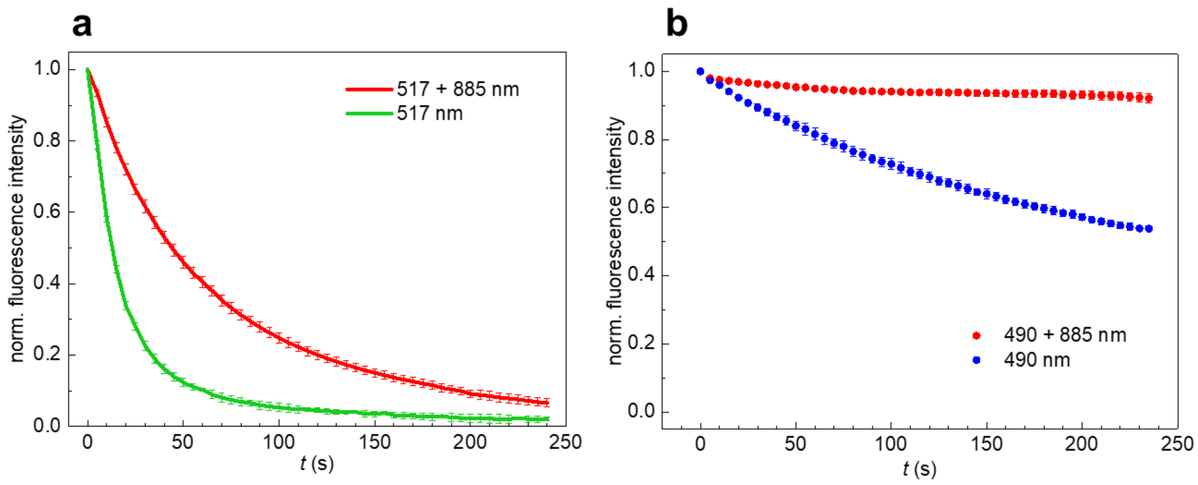

Photobleaching kinetics of live FP-labelled *E. coli* bacteria under continuous illumination by the modified Nikon microscope:

The samples were illuminated with continuous visible and NIR light and imaged every 5 s (exposure time 50 ms). **a,** YPet-labelled bacteria (*E. coli* MG1655) growing on a MMcasa-agarose pad illuminated at 517 nm (1.2 W/cm^2) alone or combined with 885-nm co-illumination (0.8 kW/cm^2). Values are mean ± s.d. (n=3 colonies from 1 sample). **b,** EGFP-labelled bacteria (*E. coli* BL21) growing on a LB-agarose pad illuminated at 490 nm (0.5 W/cm^2) alone or combined with 885-nm co-illumination (0.8 kW/cm^2). Values are mean ± s.d. (n=3 colonies from 1 sample).

Supplementary Fig. 7.

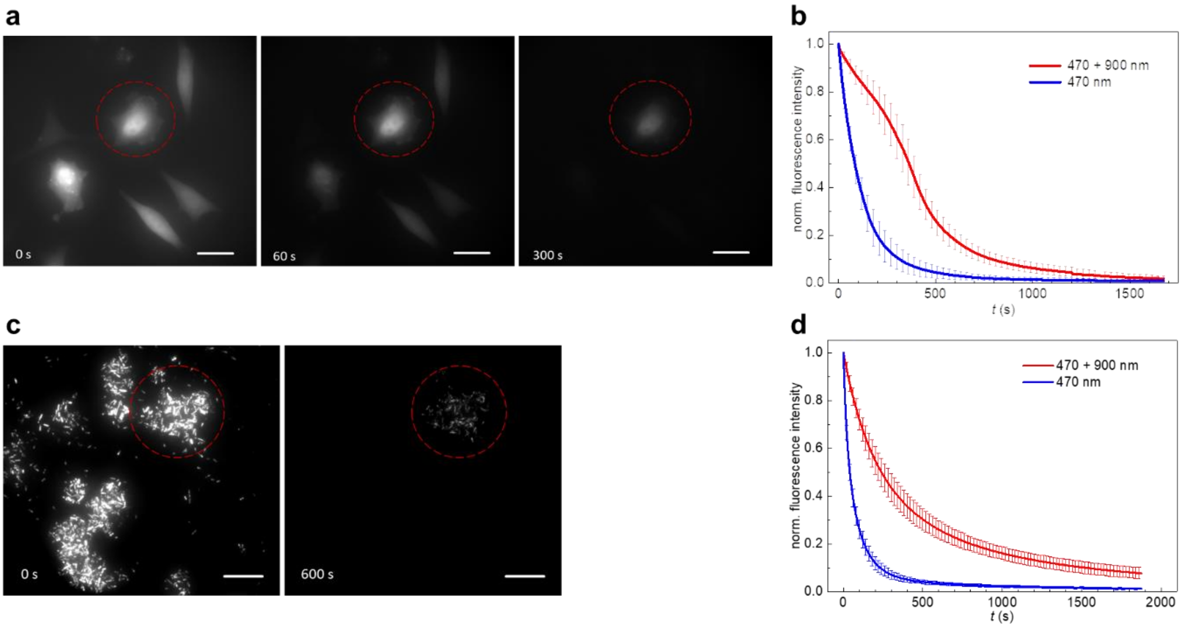

Reduced photobleaching of EGFP upon NIR co-illumination in biological samples: **a,** Fluorescence images of live EGFP-labelled HeLa cells in DMEM under continuous illumination at 470 nm (16 W/cm^2, whole field) and 900 nm (2 kW/cm^2, red dashed circle). **b,** Photobleaching kinetics of live EGFP-labelled HeLa cells illuminated solely at 470 nm (blue) or at both 470 nm and 900 nm (red). I_{470} = 16 W/cm^2. I_{900} = 2 kW/cm^2. Values are mean ± s.d. (n=8 cells from 4 samples for 470-nm illumination and n=18 cells from 4 samples for dual illumination). **c,** Fluorescence images of EGFP-labelled *E. coli* bacteria (BL21) on a PBS-agarose pad under continuous illumination at 470 nm (32 W/cm^2, whole field) and 900 nm (2 kW/cm^2, red dashed circle). **d,** Photobleaching kinetics of EGFP-labelled *E. coli* bacteria (BL21) illuminated solely at 470 nm (blue) or at both 470 nm and 900 nm (red). I_{470} = 32 W/cm^2. I_{900} = 2 kW/cm^2. Values are mean ± s.d. (n=3 samples). Scales bars in a and c: 20 µm.

Supplementary Fig. 8.

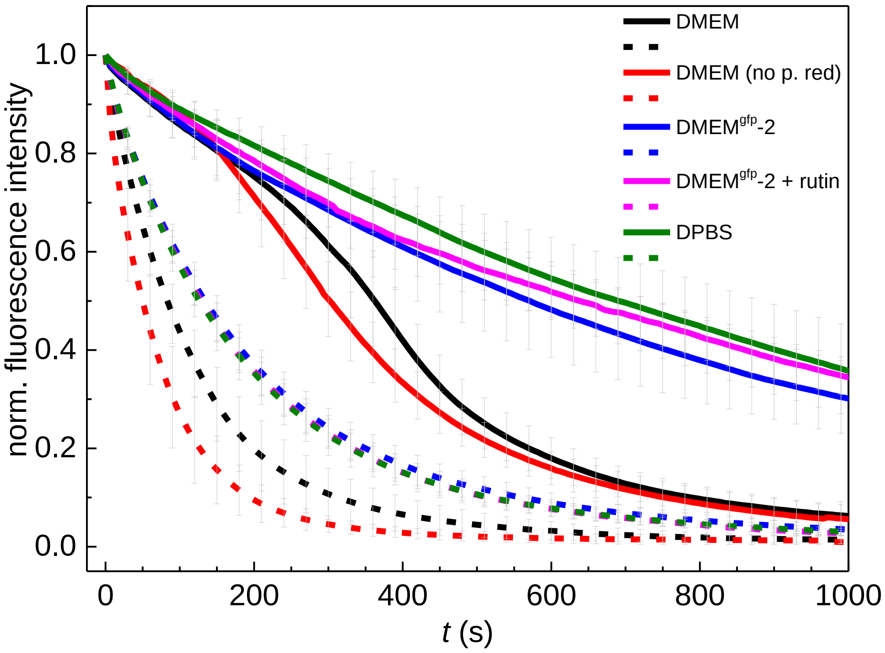

Photobleaching kinetics of EGFP-labelled live HeLa cells in different media in absence or presence of NIR co-illumination:

Continuous illumination at 470 nm (16 W/cm^2, dashed lines) alone or combined with 900-nm co-illumination (2 kW/cm^2, solid lines). Media: standard DMEM (black), DMEM without phenol red (red), DMEM^{gfp}-2 (blue), DMEM^{gfp}-2 supplemented with rutin (magenta) and DPBS (green).

For 470-nm illumination, values are mean ± s.d. (n=18 cells for standard DMEM, n=19 cells for DMEM without phenol red, n=12 cells for DMEM^{gfp}-2, n=16 cells for DMEM^{gfp}-2 + rutin and n=17 cells for DPBS, examined over 4 samples for each medium).

For dual illumination, values are mean ± s.d. (n=7 cells for standard DMEM, n=4 cells for DMEM without phenol red, n=6 cells for DMEM^{gfp}-2, n=7 cells for DMEM^{gfp}-2 + rutin and n=5 cells for DPBS, examined over 4 samples for each medium).

Supplementary Fig. 9.

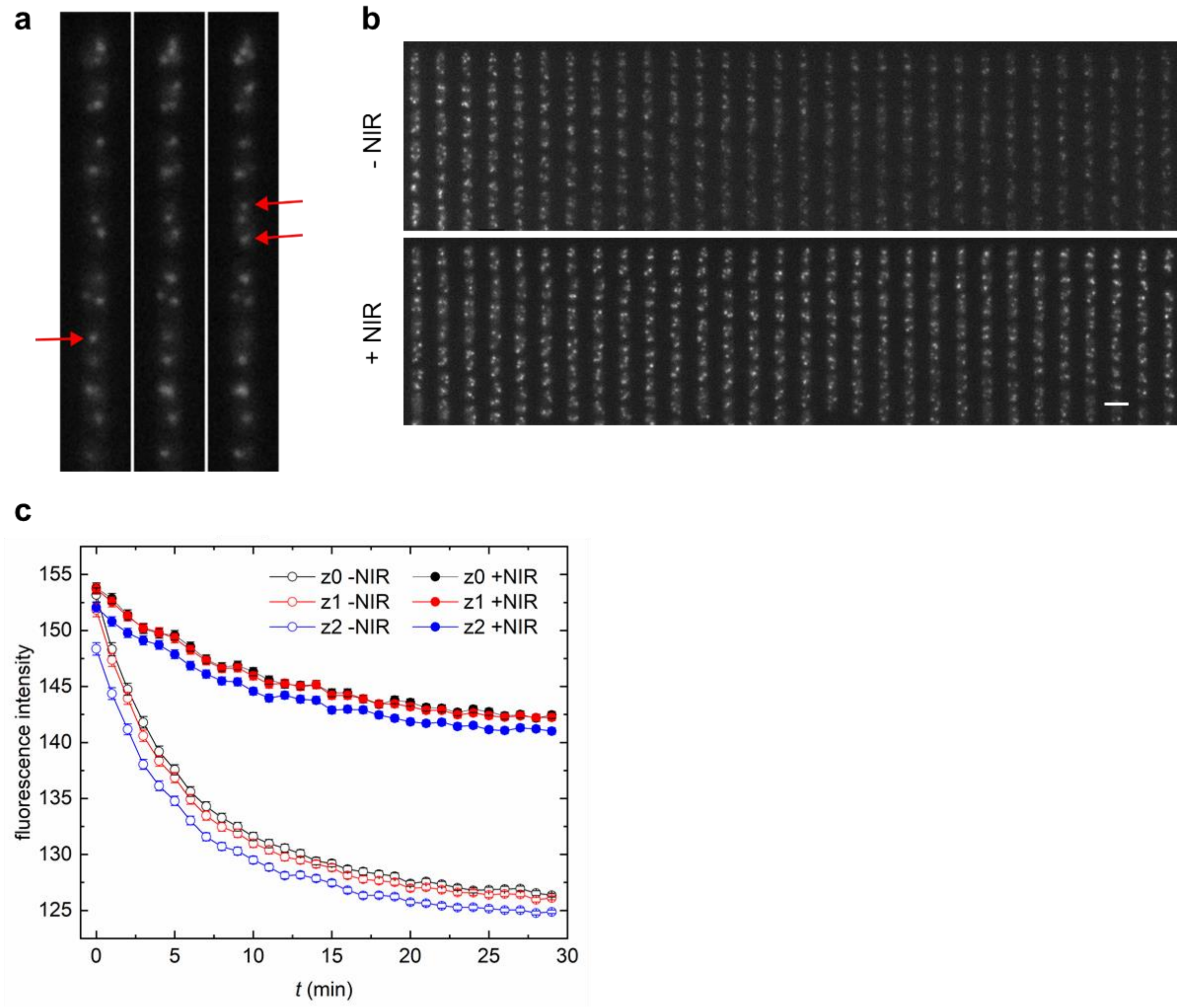

NIR co-illumination improves the tracking of YPet-labelled replisomes in *E. coli*: Replisomes were marked using a fluorescent fusion of the β-subunit of the DNA polymerase, DnaN-YPet, resulting in bright spots inside the cells. Cells were grown at 37°C in LB medium (~22 min doubling time) in a "mother machine" microfluidic chip[32] and imaged every 1 min by illumination at 517 nm (1.2 W/cm^2) alone or combined with 885 nm (0.8 kW/cm^2), at 3 different z-planes distant from 350 nm (total exposure time: 3 × 300 ms). **a**, Images of a same microfluidic channel at the 3 z-planes. Red arrows show spots that are not clearly visible in the middle plane and require the measurement of the other planes to be detected. **b**, Kymographs of microfluidic channels in the middle plane obtained for illumination at 517 nm alone (top) or combined with 885 nm (bottom) and highlighting the reduction of YPet photobleaching by NIR light. We show here all recorded images (1 per min). Scale bar: 2 µm. **c**, Time evolution of the fluorescence intensity of bacteria in the 3 z-planes (z0, z1, z2) for illumination at 517 nm alone (open circles) or combined with 885 nm (solid circles). Values are mean ± s.e.m. (n=240 cells).

Supplementary Fig. 10

First cell preparation:

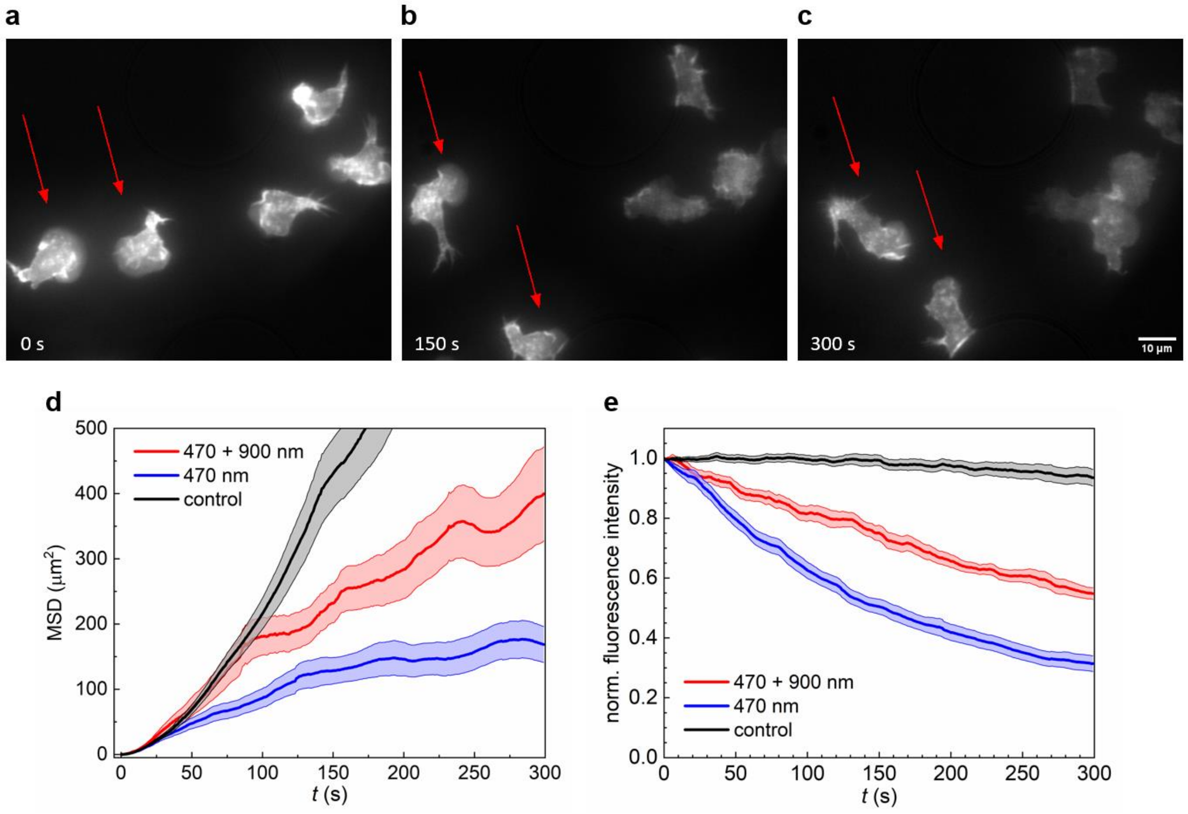

Second cell preparation:

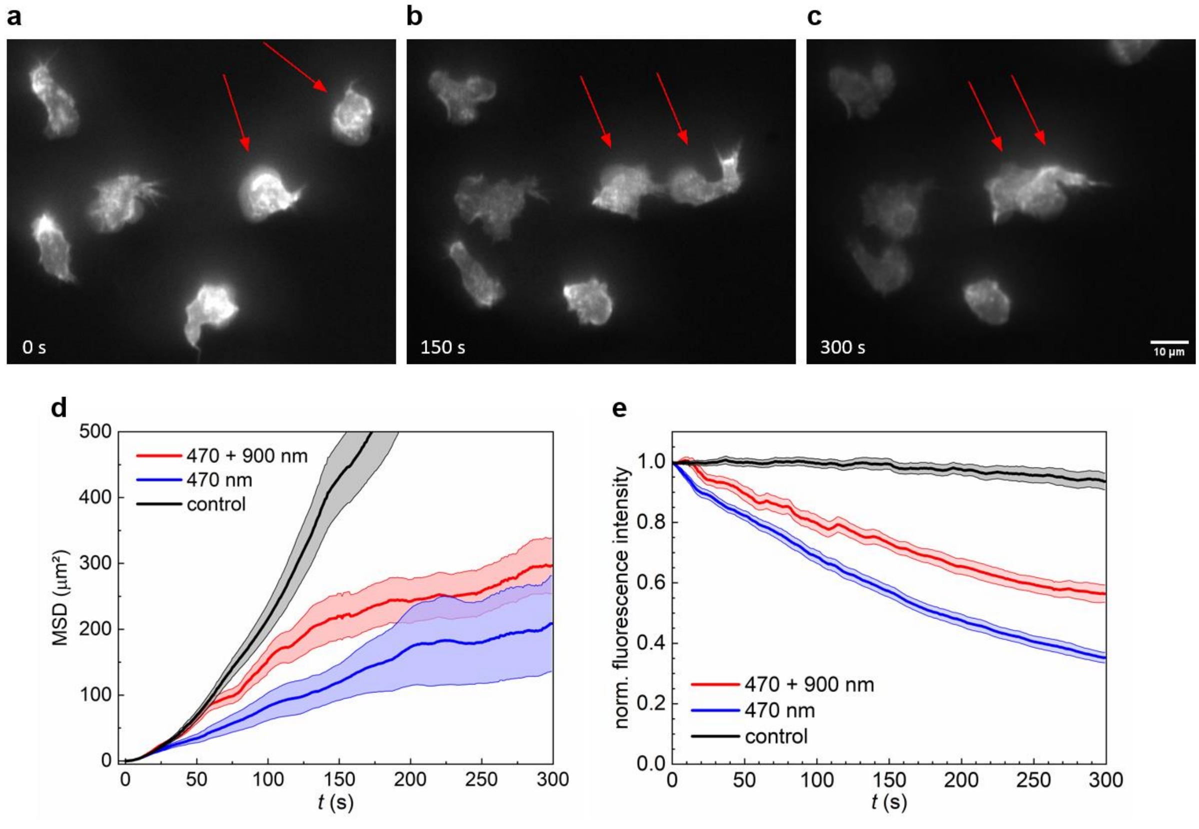

Reduction of photobleaching and phototoxicity in primary mouse neutrophils expressing LifeAct-GFP and co-illuminated with NIR light:

We show the results obtained for two cell preparations from different mice.

Neutrophils were confined in PDMS (polydimethylsiloxane) microchambers to promote their movement in 2D, and their photobleaching and motility were assessed for 5 min at 37°C under different illumination conditions: weak 470 nm (0.8 W/cm^2), strong 470 nm (20 W/cm^2) or strong 470 nm (20 W/cm^2) combined with 900-nm co-illumination (2 kW/cm^2) over part of the field of view. Control cells were exposed to weak 470 nm for 200 ms every 1 s. Other cells were exposed to strong 470 nm with or without 900-nm co-illumination for 400 ms every 1 s (first cell preparation) or for 200 ms every 1 s (second cell preparation).

a-c, Fluorescence images of neutrophils at the start, middle and end of illumination with strong 470 nm. Neutrophils indicated by the arrows were co-illuminated at 900 nm and show reduced photobleaching. Scale bars: 10 μm.

d, Mean squared displacement (MSD) of neutrophils illuminated with weak (control, black) or strong 470 nm, alone (blue) or combined with 900-nm co-illumination (red).

e, Fluorescence photobleaching curves of neutrophils illuminated with weak (control, black) or strong 470 nm, alone (blue) or combined with 900-nm co-illumination (red). All curves were normalized to 1 at t=0.

For the first cell preparation, values are mean ± s.e.m. (n=32 cells from 6 microchambers) for 470-nm illumination, mean ± s.e.m. (n=14 cells from 6 microchambers) for dual illumination and mean ± s.e.m. (n=44 cells from 5 microchambers) for control.

For the second cell preparation, values are mean ± s.e.m. (n=35 cells from 8 microchambers) for 470-nm illumination, mean ± s.e.m. (n=18 cells from 8 microchambers) for dual illumination and mean ± s.e.m. (n= 44 cells from 5 microchambers) for control.

As can be seen in panels d, the MSD of 470-nm illuminated cells (blue) was significantly lower from the onset than that of control cells (black) due to the phototoxicity of strong 470-nm light. In contrast, cells illuminated at both 470 nm and 900 nm (red) maintained MSD values similar to the control for 60-100 s. Despite a departure from control at longer times, co-illuminated cells kept on migrating further than cells illuminated only at 470 nm. These experiments show a protective effect of NIR light against phototoxicity due to visible excitation.

Supplementary Fig. 11.

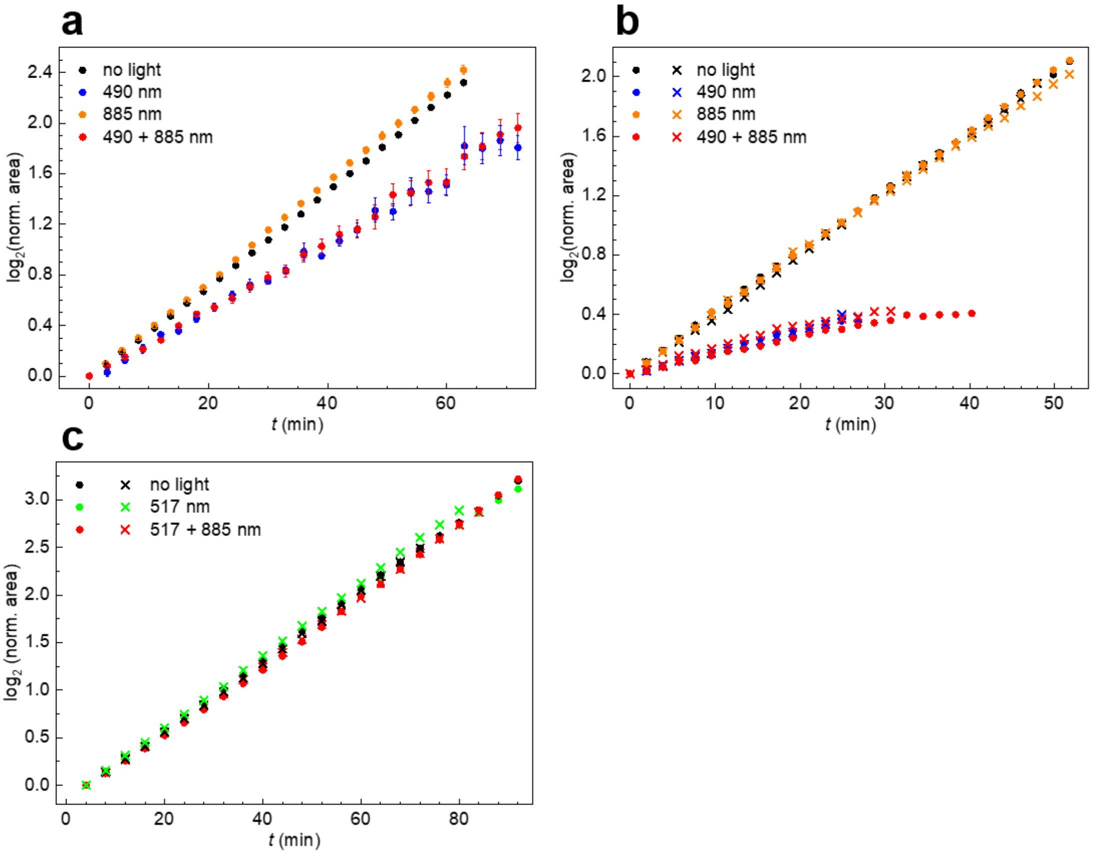

Growth of EGFP-labelled (a) and unlabelled (b, c) *E. coli* colonies subjected to different illuminations:

The growth was followed by phase-contrast imaging. We show here the temporal evolution of the binary logarithm of the area of the microcolonies normalized with respect to their initial area. **a,** EGFP-labelled colonies (BL21) on a LB-agarose pad. Unexposed colonies (black) were subjected to minimal exposures of 100 ms of low intensity white light every 3 min, for the sole purpose of recording images. Exposed colonies were illuminated for 5 s every 3 min with 490-nm light (0.5 W/cm^2, blue), 885-nm light (0.8 kW/cm^2, orange) or both (red). Values are mean ± s.e.m. (n=4 colonies from 1 sample). **b,** Unlabelled colonies (MG1655) on a LB-agarose pad. Unexposed colonies (black) were subjected to minimal exposures of 100 ms of low intensity white light every 3 min, for the sole purpose of recording images. Exposed colonies were illuminated for 5 s every 2 min with 490-nm light (0.5 W/cm^2, blue), 885-nm light (0.8 kW/cm^2, orange) or both (red). The circles and crosses show the results for two colonies from the same sample in each illumination condition. **c,** Unlabelled colonies (MG1655) on a MMcasa-agarose pad. Unexposed colonies (black) were subjected to minimal exposures of 100 ms of low intensity white light every 4 min, for the sole purpose of recording images. Exposed colonies were illuminated for 2 s every 4 min with 517-nm light (1.2 W/cm^2, blue) alone or combined with 885-nm light (0.8 kW/cm^2, red). The circles and crosses show the results for two colonies from the same sample in each illumination condition.

Note that the experiments with EGFP-labelled and unlabelled bacteria exposed to 490-nm light were performed using different *E. coli* strains. They should therefore not be directly compared. The control on unlabelled bacteria is here only intended to illustrate the toxicity of blue light to bacteria, which is well documented in the literature.[29]

Supplementary Fig. 12.

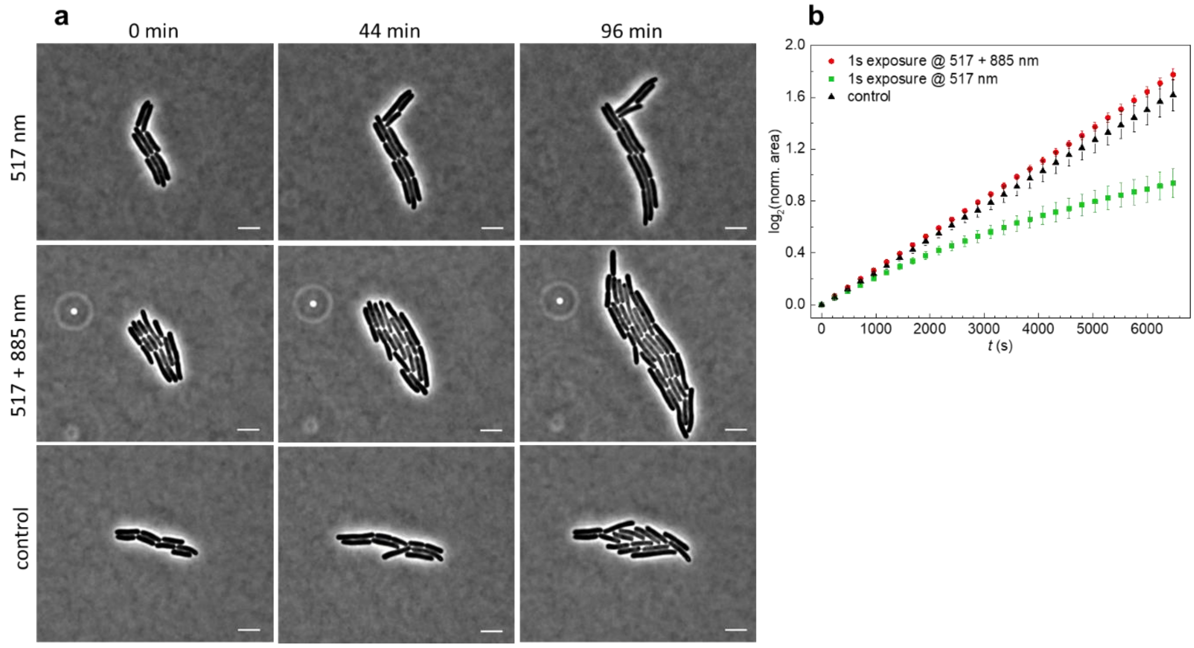

Reduction of phototoxicity in YPet-labelled *E. coli* colonies subjected to 1-s exposures to 517 and 885-nm light:
a, Phase-contrast images showing the growth of YPet-labelled *E. coli* colonies subjected to different illumination conditions: 517 nm alone (top), 517 nm and 885 nm (middle) and no illumination (bottom). I_{517} = 1.2 W/cm^2. I_{885} = 0.8 kW/cm^2. Samples were exposed to 517-nm and/or 885-nm light for 1 s every 4 min. Control colonies were minimally illuminated with a white LED (0.1s exposure) for the sole purpose of recording images. Scale bars: 5 µm. T=37°C.
b, Time evolution of the area of the bacterial colonies normalized to their initial area, in binary logarithmic scale. Values are mean ± s.e.m. (n=14 colonies (4 for control) from 2 samples).

We performed this experiment with three different exposure times to 517 and 885-nm light: 1 s, 2 s and 10 s. As shown here and in Fig. 2h-i, for 1 s and 2 s, growth is strongly affected in the absence of NIR light but completely restored in the presence of NIR light. In contrast, for 10 s of exposure, growth was strongly affected and cells completely stopped growing after 1 h even in the presence of NIR light.

Supplementary Fig. 13.

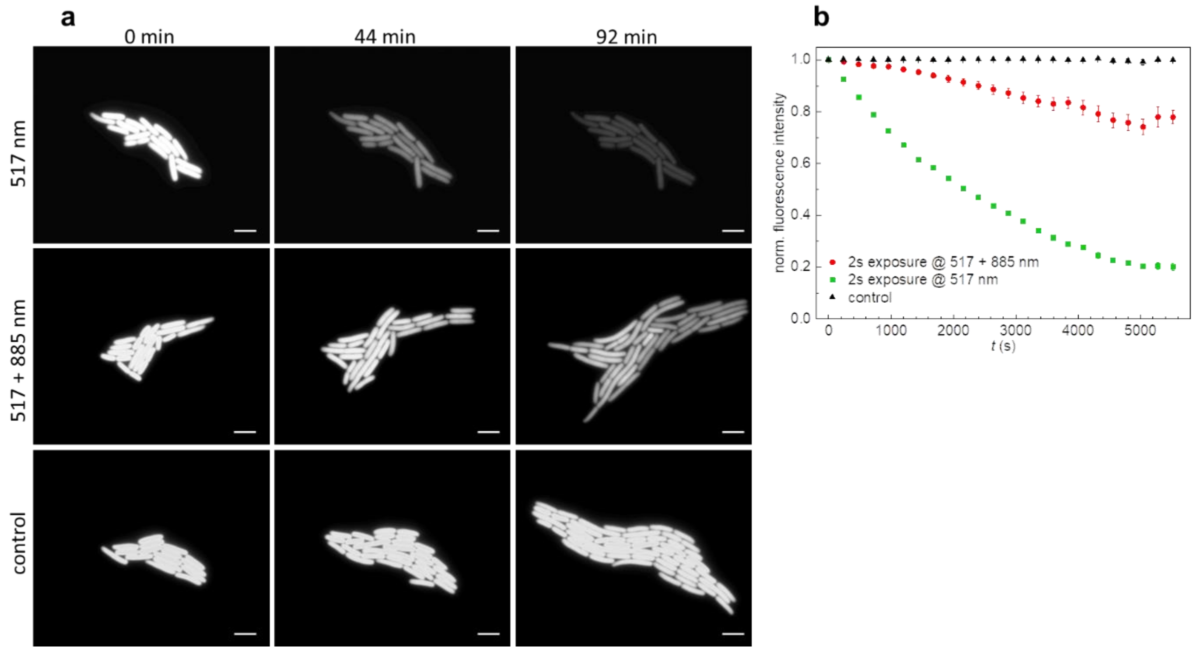

Reduction of photobleaching in YPet-labelled *E. coli* colonies subjected to 2-s exposures to 517 and 885-nm light:

a, Fluorescence images of growing YPet-labelled *E. coli* colonies subjected to different illumination conditions: 517 nm alone (top), 517 nm and 885 nm (middle) and no illumination (bottom). I_{517} = 1.2 W/cm^2. I_{885} = 0.8 kW/cm^2. Samples were exposed to 517-nm and/or 885-nm light for 2 s every 4 min. Control colonies were minimally illuminated with 517-nm light (0.1s exposure) for the sole purpose of recording images. Scale bars: 5 µm. T=37°C. **b**, Photobleaching kinetics of colonies illuminated solely at 517 nm (green squares) or at both 517 and 885 nm (red circles) and of control colonies (black triangles). Values are mean ± s.e.m. (n=14 colonies (4 for control) from 2 samples).

Supplementary Fig. 14.

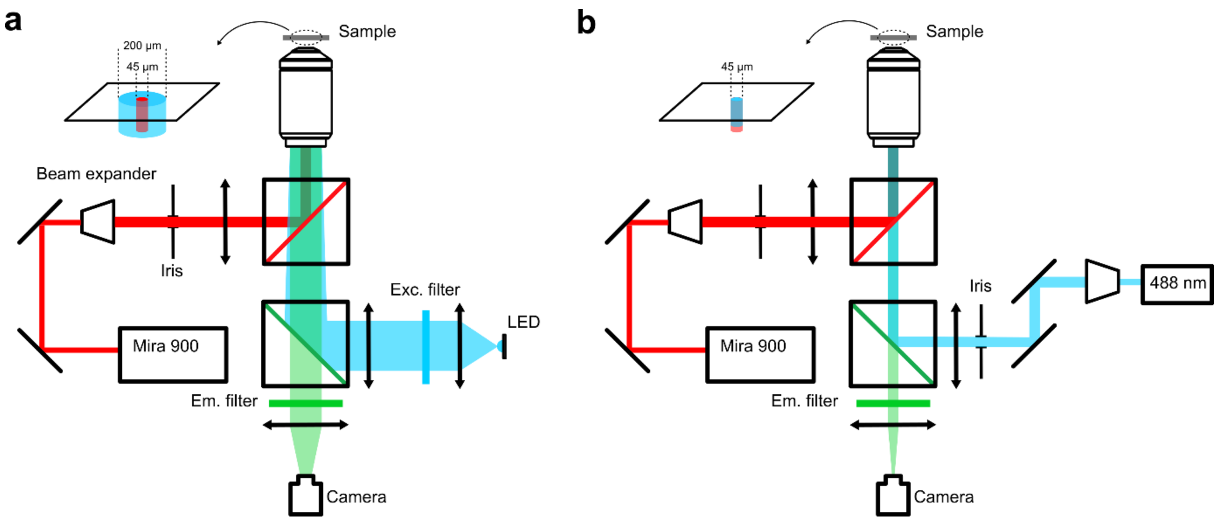

Home-built optical setups used for the characterization of the RP effect in EGFP: NIR co-illumination is delivered over a 45-µm diameter round spot by a continuous laser tunable between 700 nm and 1000 nm (Mira, Coherent). Visible excitation is provided either by a LED or by a laser diode, depending on the desired intensity. **a,** Setup with a LED as visible excitation source. Visible excitation is delivered over a 200-µm diameter round spot larger than the NIR beam, which allows the simultaneous measurement of photobleaching kinetics in the presence and absence of NIR. This setup was used to achieve visible intensities of less than 32 W/cm^2. **b,** Setup with a laser diode as visible excitation source. Visible excitation is delivered over the same 45-µm diameter spot as the NIR co-illumination. This setup was used to achieve visible intensities of more than 32 W/cm^2.

4. Supplementary tables

Supplementary Table 1.

Illumination conditions used for the different experiments.

Figure N°	λ_{VIS}	I_{VIS}	λ_{NIR}	I_{NIR}	Illumination mode
1a,b,h; 2a,b; S1; S7c,d	470 nm	32 W/cm^2	900 nm	2 kW/cm^2	continuous
1c	470 nm	32 W/cm^2	900 nm	variable[1]	continuous
1d	488 nm	variable[1]	900 nm	2 kW/cm^2	continuous
1e	470 nm	32 W/cm^2	variable[1]	20 W/cm^2	continuous
2c; S7a,b; S8	470 nm	16 W/cm^2	900 nm	2 kW/cm^2	continuous
2d,e; S9	517 nm	1.2 W/cm^2	885 nm	0.8 kW/cm^2	3×300 ms every 1 min[2]
2f; S10	470 nm	20 W/cm^2	900 nm	2 kW/cm^2	200 ms every 1 s
2g; S10	470 nm	20 W/cm^2	900 nm	2 kW/cm^2	400 ms every 1 s
2g; S10 (control)	470 nm	0.8 W/cm^2	-	-	200 ms every 1 s
2h,i; S11c; S13	517 nm	1.2 W/cm^2	885 nm	0.8 kW/cm^2	2 s every 4 min
S4	470 nm	0.2 W/cm^2	900 nm	1.7 kW/cm^2	continuous
S2	470 nm	35 W/cm^2	variable[1]	variable[1]	continuous
S3	560 nm	5.8 W/cm^2	725 nm	248 W/cm^2	continuous
S6a	517 nm	1.2 W/cm^2	885 nm	0.8 kW/cm^2	continuous
S6b	490 nm	0.5 W/cm^2	885 nm	0.8 kW/cm^2	continuous
S11a	490 nm	0.5 W/cm^2	885 nm	0.8 kW/cm^2	5 s every 3 min
S11b	490 nm	0.5 W/cm^2	885 nm	0.8 kW/cm^2	5 s every 2 min
S12	517 nm	1.2 W/cm^2	885 nm	0.8 kW/cm^2	1 s every 4 min

[1]See figure.
[2]We measured 3 successive images every minute in 3 different planes with exposure times of 300 ms.

Supplementary Table 2.

Cloning of the bacterial expression vectors of sfGFP, acGFP, mWasabi and mClover.

Primer	Sequence (5' to 3')	Template
ag321	gccctgaaaatacaggttttcGCTAGC	pET28a
Kan-F	GCATCAACCAAACCGTTATTCATTCGTG	
ag322	TAATAGCTCGAGCACCACCACCAC	pET28a
Kan-R	CACGAATGAATAACGGTTTGGTTGATGC	

G-block	Sequence
sfGFP	GCTAGCgaaaacctgtattttcagggcatggtgagcaagggcgaggagctgttcaccggggtggtgcccatcctggtcgagctggacggcgacgtaaacggccacaagttcagcgtgcgcggcgagggcgagggcgatgccaccaacggcaagctgaccctgaagttcatctgcaccaccggcaagctgcccgtgccctggcccaccctcgtgaccaccctgacctacggcgtgcagtgcttcagccgctaccccgaccacatgaagcgccacgacttcttcaagtccgccatgcccgaaggctacgtccaggagcgcaccatcagcttcaaggacgacggcacctacaagacccgcgccgaggtgaagttcgagggcgacaccctggtgaaccgcatcgagctgaagggcatcgacttcaaggaggacggcaacatcctggggcacaagctggagtacaacttcaacagccacaacgtctatatcaccgccgacaagcagaagaacggcatcaaggccaacttcaagatccgccacaacgtggaggacggcagcgtgcagctcgccgaccactaccagcagaacacccccatcggcgacggccccgtgctgctgcccgacaaccactacctgagcacccagtccgtgctgagcaaagaccccaacgagaagcgcgatcacatggtcctgctggagttcgtgaccgccgccgggatcactcacggcatggacgagctgtacaagtaaTAGCTCGAGCACCACCACCAC
acGFP	GCTAGCgaaaacctgtattttcagggcatggtgagcaagggcgccgagctgttcaccggcatcgtgcccatcctgatcgagctgaatggcgatgtgaatggccacaagttcagcgtgagcggcgagggcgagggcgatgccacctacggcaagctgaccctgaagttcatctgcaccaccggcaagctgcctgtgccctggcccaccctggtgaccaccctgagctacggcgtgcagtgcttctcacgctaccccgatcacatgaagcagcacgacttcttcaagagcgccatgcctgagggctacatccaggagcgcaccatcttcttcgaggatgacggcaactacaagtcgcgcgccgaggtgaagttcgagggcgataccctggtgaatcgcatcgagctgaccggcaccgatttcaaggaggatggcaacatcctgggcaataagatggagtacaactacaacgcccacaatgtgtacatcatgaccgacaaggccaagaatggcatcaaggtgaacttcaagatccgccacaacatcgaggatggcagcgtgcagctggccgaccactaccagcagaatacccccatcggcgatggccctgtgctgctgcccgataaccactacctgtccacccagagcgccctgtccaaggaccccaacgagaagcgcgatcacatgatctacttcggcttcgtgaccgccgccgccatcacccacggcatggatgagctgtacaagtaaTAGCTCGAGCACCACCACCAC
mWasabi	GCTAGCgaaaacctgtattttcagggcatggtgagcaagggcgaggagaccacaatgggcgtaatcaagcccgacatgaagatcaagctgaagatggagggcaacgtgaatggccacgccttcgtgatcgagggcgagggcgagggcaagccctacgacggcaccaacaccatcaacctggaggtgaaggagggagccccccctgcccttctcctacgacattctgaccaccgcgttcagttacggcaacagggccttcaccaagtaccccgacgacatccccaactacttcaagcagtccttccccgagggctactcttgggagcgcaccatgaccttcgaggacaagggcatcgtgaaggtgaagtccgacatctccatggaggaggactccttcatctacgagatacacctcaagggcgagaacttcccccccaacggccccgtgatgcagaaggagaccaccggctgggacgcctccaccgagaggatgtacgtgcgcgacggcgtgctgaagggcgacgtcaagatgaagctgctgctggagggcggcggccaccaccgcgttgacttcaagaccatctacagggccaagaaggcggtgaagctgcccgactatcactttgtggaccaccgcatcgagatcctgaaccacgacaaggactacaacaaggtgaccgtttacgagatcgccgtggcccgcaactccaccgacggcatggacgagctgtacaagtaaTAGCTCGAGCACCACCACCAC
mClover	GCTAGCgaaaacctgtattttcagggcatggtgagcaagggcgaggagctgttcaccggggtggtgcccatcctggtcgagctggacggcgacgtaaacggccacaagttcagcgtccgcggcgagggcgagggcgatgccaccaacggcaagctgaccctgaagttcatctgcaccaccggcaagctgcccgtgccctggcccaccctcgtgaccaccttcggctacggcgtggcctgcttcagccgctaccccgaccacatgaagcagcacgacttcttcaagtccgccatgcccgaaggctacgtccaggagcgcaccatctctttcaaggacgacggtacctacaagacccgcgccgaggtgaagttcgagggcgacaccctggtgaaccgcatcgagctgaagggcatcgacttcaaggaggacggcaacatcctggggcacaagctggagtacaacttcaacagccacaacgtctatatcacggccgacaagcagaagaacggcatcaaggctaacttcaagatccgccacaacgttgaggacggcagcgtgcagctcgccgaccactaccagcagaacacccccatcggcgacggccccgtgctgctgcccgacaaccactacctgagccatcagtccgccctgagcaaagaccccaacgagaagcgcgatcacatggtcctgctggagttcgtgaccgccgccgggattacacatggcatggacgagctgtacaagtaaTAGCTCGAGCACCACCACCAC

5. Supplementary video captions

Supplementary Video 1.

NIR co-illumination reduces the photobleaching of live HeLa cells labelled with EGFP:
This fluorescence movie shows the photobleaching of live HeLa cells labelled with EGFP in DMEM. All cells were illuminated at 470 nm. Cells inside the red circle were in addition co-illuminated at 900 nm. I_{470} = 38 W/cm^2. I_{900} = 1.8 kW/cm^2. Both illuminations were applied continuously. Cells illuminated at both 470 nm and 900 nm show slower photobleaching than cells illuminated only at 470 nm.

Supplementary Video 2.

NIR co-illumination extends the visualization of YPet-labelled replisomes in rapidly growing *E. coli* cells:
This fluorescence movie shows *E. coli* cells growing in "mother machine" microfluidic chips at 37°C in LB medium (~22-min doubling time). The replisomes are marked using a fluorescent fusion of the β-subunit of the DNA polymerase, DnaN-YPet, resulting in bright spots inside the cells. The spots quickly disappear in the chip illuminated only at 517 nm due to YPet photobleaching. In contrast, they are still perfectly visible after one cell cycle in the chip illuminated at both 517 nm and 885 nm. The chips were imaged every 1 min by illumination at 517 nm (1.2 W/cm^2) alone or combined with 885 nm (0.8 kW/cm^2), at 3 different z-planes distant from 350 nm (total exposure time: 3 × 300 ms) to allow detection of all replisomes. Here we show only the middle plane.

Supplementary Video 3.

NIR co-illumination reduces photobleaching and phototoxicity in primary mouse neutrophils expressing LifeAct-GFP:
This fluorescence movie shows primary mouse neutrophils expressing LifeAct-GFP, migrating in a PDMS microchamber at 37°C. All cells were illuminated at 470 nm. The two cells indicated by the red arrows were in addition co-illuminated at 900 nm. I_{470} = 20 W/cm^2. I_{900} = 2 kW/cm^2. Cells were exposed to 470-nm and 900-nm light for 200 ms every 1 s. Cells illuminated at both 470 nm and 900 nm show lower photobleaching and higher motility than cells illuminated only at 470 nm.

Supplementary Video 4.

NIR co-illumination reduces phototoxicity in YPet-labelled *E. coli* cells:
This phase-contrast movie shows the growth of YPet-labelled *E. coli* microcolonies on agarose pads at 37°C, under different illumination conditions: 517 nm alone (left), 517 nm and 885 nm (middle) and no illumination (right). I_{517} = 1.2 W/cm^2. I_{885} = 0.8 kW/cm^2. The samples were exposed to 517-nm and/or 885-nm light for 2 s every 4 min. The phase-contrast images were obtained with white light of negligible intensity. The growth of microcolonies illuminated only at 517 nm is almost stopped, while microcolonies illuminated at both 517 nm and 885 nm show a growth rate similar to that of the non-illuminated control.

References:

1 Byrdin, M., Duan, C., Bourgeois, D. & Brettel, K. A Long-Lived Triplet State Is the Entrance Gateway to Oxidative Photochemistry in Green Fluorescent Proteins. *J. Am. Chem. Soc.* **140**, 2897-2905 (2018).

2 Ringemann, C., Schönle, A., Giske, A., von Middendorff, C., Hell, S. W. & Eggeling, C. Enhancing fluorescence brightness: effect of reverse intersystem crossing studied by fluorescence fluctuation spectroscopy. *ChemPhysChem* **9**, 612-624 (2008).

3 Sarkisyan, K. S., Goryashchenko, A. S., Lidsky, P. V., Gorbachev, D. A., Bozhanova, N. G., Gorokhovatsky, A. Y., Pereverzeva, A. R., Ryumina, A. P., Zherdeva, V. V., Savitsky, A. P., Solntsev, K. M., Bommarius, A. S., Sharonov, G. V., Lindquist, J. R., Drobizhev, M., Hughes, T. E., Rebane, A., Lukyanov, K. A. & Mishin, A. S. Green fluorescent protein with anionic tryptophan-based chromophore and long fluorescence lifetime. *Biophys. J.* **109**, 380-389 (2015).

4 Klan, P. & Wirz, J. *Photochemistry of organic compounds: from concepts to practice*. (Wiley, 2009).

5 Acharya, A., Bogdanov, A. M., Grigorenko, B. L., Bravaya, K. B., Nemukhin, A. V., Lukyanov, K. A. & Krylov, A. I. Photoinduced Chemistry in Fluorescent Proteins: Curse or Blessing? *Chem. Rev.* **117**, 758-795 (2017).

6 Tsien, R. Y. The green fluorescent protein. *Annu. Rev. Biochem.* **67**, 509-544 (1998).

7 Zimmer, M. Green fluorescent protein (GFP): Applications, structure, and related photophysical behavior. *Chem. Rev.* **102**, 759-781 (2002).

8 Dickson, R. M., Cubitt, A. B., Tsien, R. Y. & Moerner, W. E. On/off blinking and switching behaviour of single molecules of green fluorescent protein. *Nature* **388**, 355-358 (1997).

9 Garcia-Parajo, M. F., Segers-Nolten, G. M., Veerman, J. A., Greve, J. & van Hulst, N. F. Real-time light-driven dynamics of the fluorescence emission in single green fluorescent protein molecules. *Proc. Natl. Acad. Sci. U. S. A.* **97**, 7237-7242 (2000).

10 Sinnecker, D., Voigt, P., Hellwig, N. & Schaefer, M. Reversible photobleaching of enhanced green fluorescent proteins. *Biochemistry* **44**, 7085-7094 (2005).

11 De Zitter, E., Thedie, D., Monkemoller, V., Hugelier, S., Beaudouin, J., Adam, V., Byrdin, M., Van Meervelt, L., Dedecker, P. & Bourgeois, D. Mechanistic investigation of mEos4b reveals a strategy to reduce track interruptions in sptPALM. *Nat. Methods* **16**, 707-710 (2019).

12 Brakemann, T., Stiel, A. C., Weber, G., Andresen, M., Testa, I., Grotjohann, T., Leutenegger, M., Plessmann, U., Urlaub, H., Eggeling, C., Wahl, M. C., Hell, S. W. & Jakobs, S. A reversibly photoswitchable GFP-like protein with fluorescence excitation decoupled from switching. *Nat. Biotechnol.* **29**, 942-947 (2011).

13 Bogdanov, A. M., Mishin, A. S., Yampolsky, I. V., Belousov, V. V., Chudakov, D. M., Subach, F. V., Verkhusha, V. V., Lukyanov, S. & Lukyanov, K. A. Green fluorescent proteins are light-induced electron donors. *Nat. Chem. Biol.* **5**, 459-461 (2009).

14 Redmond, R. W., Kochevar, I. E., Krieg, M., Smith, G. & McGimpsey, W. G. Excited-state relaxation in cyanine dyes: a remarkably efficient reverse intersystem crossing from upper triplet levels. *J. Phys. Chem. A* **101**, 2773-2777 (1997).

15 Reindl, S. & Penzkofer, A. Higher excited-state triplet-singlet intersystem crossing of some organic dyes. *Chem. Phys.* **211**, 431-439 (1996).

16 Song, L., Varma, C. A., Verhoeven, J. W. & Tanke, H. J. Influence of the triplet excited state on the photobleaching kinetics of fluorescein in microscopy. *Biophys. J.* **70**, 2959-2968 (1996).

17 Lougnot, D.-J. & Goldschmidt, C. R. Photoionization of fluorescein via excited triplet and singlet states. *J. Photochem.* **12**, 215-224 (1980).

18 Bittel, A. M., Nickerson, A., Saldivar, I. S., Dolman, N. J., Nan, X. & Gibbs, S. L. Methodology for Quantitative Characterization of Fluorophore Photoswitching to Predict Superresolution Microscopy Image Quality. *Sci. Rep.* **6**, 29687 (2016).

19 Icha, J., Weber, M., Waters, J. C. & Norden, C. Phototoxicity in live fluorescence microscopy, and how to avoid it. *Bioessays* **39** (2017).

20 Hockberger, P. E., Skimina, T. A., Centonze, V. E., Lavin, C., Chu, S., Dadras, S., Reddy, J. K. & White, J. G. Activation of flavin-containing oxidases underlies light-induced production of H2O2 in mammalian cells. *Proc. Natl. Acad. Sci. U. S. A.* **96**, 6255-6260 (1999).

21 Gwynne, P. J. & Gallagher, M. P. Light as a Broad-Spectrum Antimicrobial. *Front. Microbiol.* **9**, 119 (2018).

22 Waldchen, S., Lehmann, J., Klein, T., van de Linde, S. & Sauer, M. Light-induced cell damage in live-cell super-resolution microscopy. *Sci. Rep.* **5**, 15348 (2015).

23 Huhner, J., Ingles-Prieto, A., Neususs, C., Lammerhofer, M. & Janovjak, H. Quantification of riboflavin, flavin mononucleotide, and flavin adenine dinucleotide in mammalian model cells by CE with LED-induced fluorescence detection. *Electrophoresis* **36**, 518-525 (2015).

24 Cranfill, P. J., Sell, B. R., Baird, M. A., Allen, J. R., Lavagnino, Z., de Gruiter, H. M., Kremers, G. J., Davidson, M. W., Ustione, A. & Piston, D. W. Quantitative assessment of fluorescent proteins. *Nat. Methods* **13**, 557-562 (2016).

25 Shaner, N. C., Steinbach, P. A. & Tsien, R. Y. A guide to choosing fluorescent proteins. *Nat. Methods* **2**, 905-909 (2005).

26 Mamontova, A. V., Grigoryev, A. P., Tsarkova, A. S., Lukyanov, K. A. & Bogdanov, A. M. Struggle for photostability: bleaching mechanisms of fluorescent proteins. *Russ. J. Bioorg. Chem.* **43**, 625-633 (2017).

27 Duan, C., Adam, V., Byrdin, M., Ridard, J., Kieffer-Jaquinod, S., Morlot, C., Arcizet, D., Demachy, I. & Bourgeois, D. Structural evidence for a two-regime photobleaching mechanism in a reversibly switchable fluorescent protein. *J. Am. Chem. Soc.* **135**, 15841-15850 (2013).

28 Melo, T. B., Ionescu, M. A., Haggquist, G. W. & Naqvi, K. R. Hydrogen abstraction by triplet flavins. I: time-resolved multi-channel absorption spectra of flash-irradiated riboflavin solutions in water. *Spectroc. Acta A* **55**, 2299-2307 (1999).

29 Leanse, L. G., Dos Anjos, C., Mushtaq, S. & Dai, T. Antimicrobial blue light: A 'Magic Bullet' for the 21st century and beyond? *Adv. Drug Deliv. Rev.* **180**, 114057 (2022).

30 Ronneberger, O., Fischer, P. & Brox, T. U-Net: Convolutional Networks for Biomedical Image Segmentation. *Lect. Notes Comput. Sc.* **9351**, 234-241 (2015).

31 Barilero, T., Le Saux, T., Gosse, C. & Jullien, L. Fluorescent Thermometers for Dual-Emission-Wavelength Measurements: Molecular Engineering and Application to Thermal Imaging in a Microsystem. *Anal. Chem.* **81**, 7988-8000 (2009).

32 Wang, P., Robert, L., Pelletier, J., Dang, W. L., Taddei, F., Wright, A. & Jun, S. Robust growth of Escherichia coli. *Curr. Biol.* **20**, 1099-1103 (2010).
