# Supplementary material for: Near-infrared co-illumination of fluorescent proteins reduces photobleaching and phototoxicity
Source: Nat Biotechnol. 2023 Aug 3;42(6):872–6. doi: 10.1038/s41587-023-01893-7 (PMC11180605; doi:10.1038/s41587-023-01893-7)
Supplement: Supplementary file 2 — Reporting Summary [file 41587_2023_1893_MOESM2_ESM.pdf]

nature research

Corresponding author(s): Agathe Espagne
Thomas Le Saux
Lydia Robert

Last updated by author(s): Jun 29, 2023

Reporting Summary

Nature Research wishes to improve the reproducibility of the work that we publish. This form provides structure for consistency and transparency in reporting. For further information on Nature Research policies, see our Editorial Policies and the Editorial Policy Checklist.

Statistics

For all statistical analyses, confirm that the following items are present in the figure legend, table legend, main text, or Methods section.

n/a	Confirmed	
☐	☒	The exact sample size (*n*) for each experimental group/condition, given as a discrete number and unit of measurement
☐	☒	A statement on whether measurements were taken from distinct samples or whether the same sample was measured repeatedly
☒	☐	The statistical test(s) used AND whether they are one- or two-sided<br>*Only common tests should be described solely by name; describe more complex techniques in the Methods section.*
☒	☐	A description of all covariates tested
☐	☒	A description of any assumptions or corrections, such as tests of normality and adjustment for multiple comparisons
☐	☒	A full description of the statistical parameters including central tendency (e.g. means) or other basic estimates (e.g. regression coefficient) AND variation (e.g. standard deviation) or associated estimates of uncertainty (e.g. confidence intervals)
☒	☐	For null hypothesis testing, the test statistic (e.g. *F*, *t*, *r*) with confidence intervals, effect sizes, degrees of freedom and *P* value noted<br>*Give P values as exact values whenever suitable.*
☒	☐	For Bayesian analysis, information on the choice of priors and Markov chain Monte Carlo settings
☒	☐	For hierarchical and complex designs, identification of the appropriate level for tests and full reporting of outcomes
☒	☐	Estimates of effect sizes (e.g. Cohen's *d*, Pearson's *r*), indicating how they were calculated

Our web collection on statistics for biologists contains articles on many of the points above.

Software and code

Policy information about availability of computer code

Data collection	Fluorescence data aimed at the mechanistic investigation of reduced photobleaching and its quantification in different samples (Fig. 1 a-e and h and Fig. 2 a-c), neutrophil data (Fig. 2 f-g) and temperature data shown in SI were collected using a home-built microscope equipped with a camera controlled by µManager 2.0. Fluorescence and phase-contrast images of bacteria (Fig. 2 d-e and h-i) were acquired using a Nikon microscope controlled by NIS software.
Data analysis	Photobleaching kinetics and the areas of growing bacterial microcolonies were extracted from the fluorescence or phase-contrast images using Fiji (ImageJ 1.53t). Further data analysis was performed with OriginPro 2015. Photophysical simulations were performed with Wolfram Mathematica 12. Temperature data were analyzed using Fiji (ImageJ 1.53t) and Matlab R2022a. Data on E. coli replisomes were analyzed with the BACMMAN software which is already published. Neutrophils were segmented and tracked using BACMMAN and further analysis was carried out with Python 3.10.

For manuscripts utilizing custom algorithms or software that are central to the research but not yet described in published literature, software must be made available to editors and reviewers. We strongly encourage code deposition in a community repository (e.g. GitHub). See the Nature Research guidelines for submitting code & software for further information.

Data

Policy information about availability of data

All manuscripts must include a data availability statement. This statement should provide the following information, where applicable:

- Accession codes, unique identifiers, or web links for publicly available datasets
- A list of figures that have associated raw data
- A description of any restrictions on data availability

The data that support the findings of this study are available at: https://doi.org/10.5281/zenodo.8069922.

Field-specific reporting

Please select the one below that is the best fit for your research. If you are not sure, read the appropriate sections before making your selection.

☒ Life sciences ☐ Behavioural & social sciences ☐ Ecological, evolutionary & environmental sciences

For a reference copy of the document with all sections, see nature.com/documents/nr-reporting-summary-flat.pdf

Life sciences study design

All studies must disclose on these points even when the disclosure is negative.

Sample size	Sample sizes are given in the figure legends. No statistical method was used to pre-determine them. They were experimentally limited by 3 factors: the impossibility of tracking multiple fields of view in parallel with the manual stage of our in-house microscope, the limited area illuminated by the NIR laser which typically covered only 1 to 3 mammalian cells at a time, and the duration of around 30 min required to photobleach EGFP completely. Taking these limitations into account, we chose sample sizes that were sufficient to show significant differences in photobleaching or phototoxicity in the different illumination conditions or imaging media tested, as indicated by the error bars.
Data exclusions	No data were excluded.
Replication	The experiment in Fig. 1a-b was performed in quadruplicate and those in Fig. 1c-d and Fig. 1h in triplicate. The experiment in Fig. 2a-b was performed in triplicate, that in Fig. 2c in quadruplicate and those in Fig. 2d-e, Fig. 2f-g and Fig. 2h-i in duplicate. All attempts at replication were successful. The consistency between results obtained in two different laboratories (PASTEUR and LJP) with different setups and light sources also supports the reproducibility of our experimental findings.
Randomization	Samples allocation into experimental groups was random. Fields of view in EGFP-PAA gels were randomly chosen to test each illumination condition. Live HeLa cells samples were randomly assigned to the different imaging media. Bacterial and mammalian cells exposed to different illumination conditions were randomly chosen within the samples.
Blinding	The same data acquisition and analysis protocols were applied to all samples regardless of their group. Blinding was not possible because sample preparation and data acquisition and analysis were performed by the same person.

Reporting for specific materials, systems and methods

We require information from authors about some types of materials, experimental systems and methods used in many studies. Here, indicate whether each material, system or method listed is relevant to your study. If you are not sure if a list item applies to your research, read the appropriate section before selecting a response.

Materials & experimental systems

n/a	Involved in the study	
☒	☐	Antibodies
☐	☒	Eukaryotic cell lines
☒	☐	Palaeontology and archaeology
☐	☒	Animals and other organisms
☒	☐	Human research participants
☒	☐	Clinical data
☒	☐	Dual use research of concern

Methods

n/a	Involved in the study	
☒	☐	ChIP-seq
☒	☐	Flow cytometry
☒	☐	MRI-based neuroimaging

Eukaryotic cell lines

Policy information about cell lines

Cell line source(s)	Hela from ATCC, cell line CCL-2, lot number 63226283
Authentication	The cell line was authenticated by STR profiling.
Mycoplasma contamination	The cell line tested negative for mycoplasma contamination.
Commonly misidentified lines (See ICLAC register)	No misidentified cell lines were used.

Animals and other organisms

Policy information about studies involving animals; ARRIVE guidelines recommended for reporting animal research

Laboratory animals	C57Bl6/J LifeaAct-GFP mice from healthy 12-week-old males.
Wild animals	The study did not involve wild animals.
Field-collected samples	The study did not involve samples collected from the field.
Ethics oversight	The present experiments, which used mouse strains exhibiting non-harmful phenotypes, did not require a project authorization and benefited from guidance of the Animal Welfare Body, Research Centre, Institut Curie.

Note that full information on the approval of the study protocol must also be provided in the manuscript.
